# Heterologous Production of Fungal Maleidrides Reveals the Cryptic Cyclization Involved in their Biosynthesis

**DOI:** 10.1002/anie.201511882

**Published:** 2016-04-21

**Authors:** Katherine Williams, Agnieszka J. Szwalbe, Nicholas P. Mulholland, Jason L. Vincent, Andrew M. Bailey, Christine L. Willis, Thomas J. Simpson, Russell J. Cox

**Affiliations:** ^1^ Institute for Organic ChemistryLeibniz University of HannoverSchneiderberg 1B30167Germany; ^2^ School of ChemistryUniversity of BristolCantock's CloseBristolBS8 1TSUK; ^3^ SyngentaJealott's HillBracknellBerkshireRG42 6EYUK; ^4^ School of Biological SciencesBristol Life Sciences BuildingUniversity of Bristol24 Tyndall AveBristolBS8 1THUK

**Keywords:** biosynthesis, cyclization, enzymes, maleidride, polyketides

## Abstract

Fungal maleidrides are an important family of bioactive secondary metabolites that consist of 7, 8, or 9‐membered carbocycles with one or two fused maleic anhydride moieties. The biosynthesis of byssochlamic acid (a nonadride) and agnestadride A (a heptadride) was investigated through gene disruption and heterologous expression experiments. The results reveal that the precursors for cyclization are formed by an iterative highly reducing fungal polyketide synthase supported by a hydrolase, together with two citrate‐processing enzymes. The enigmatic ring formation is catalyzed by two proteins with homology to ketosteroid isomerases, and assisted by two proteins with homology to phosphatidylethanolamine‐binding proteins.

Maleidrides are bioactive medium‐ring carbocyclic compounds with one or two maleic anhydride moieties and are produced by fungi. Byssochlamic acid (**1**; Figure 1), discovered by Raistrick and Smith in the 1930s,^1^ was one of the first maleidrides to have its structure elucidated in the 1960s.^2^, ^3^, ^4^ The structures of its isomers glaucanic acid (**2**)^3^ and heveadride (**3**)^5^ were also solved at that time. More recent examples include phomoidride A (**4**, an inhibitor of squalene synthase and Ras farnesyl transferase),^6^ rubratoxins such as **5** (recently shown to suppress cancer metastasis),^7^ cornexistin (**6**, which shows potent and selective herbicidal activity),^8^ and zopfiellin (**7**).^9^ Heptadrides are also known, for example, agnestadride A (**8**), which was isolated along with byssochlamic acid (**1**) from *Byssochlamys fulva*.^10^

**Figure 1:**
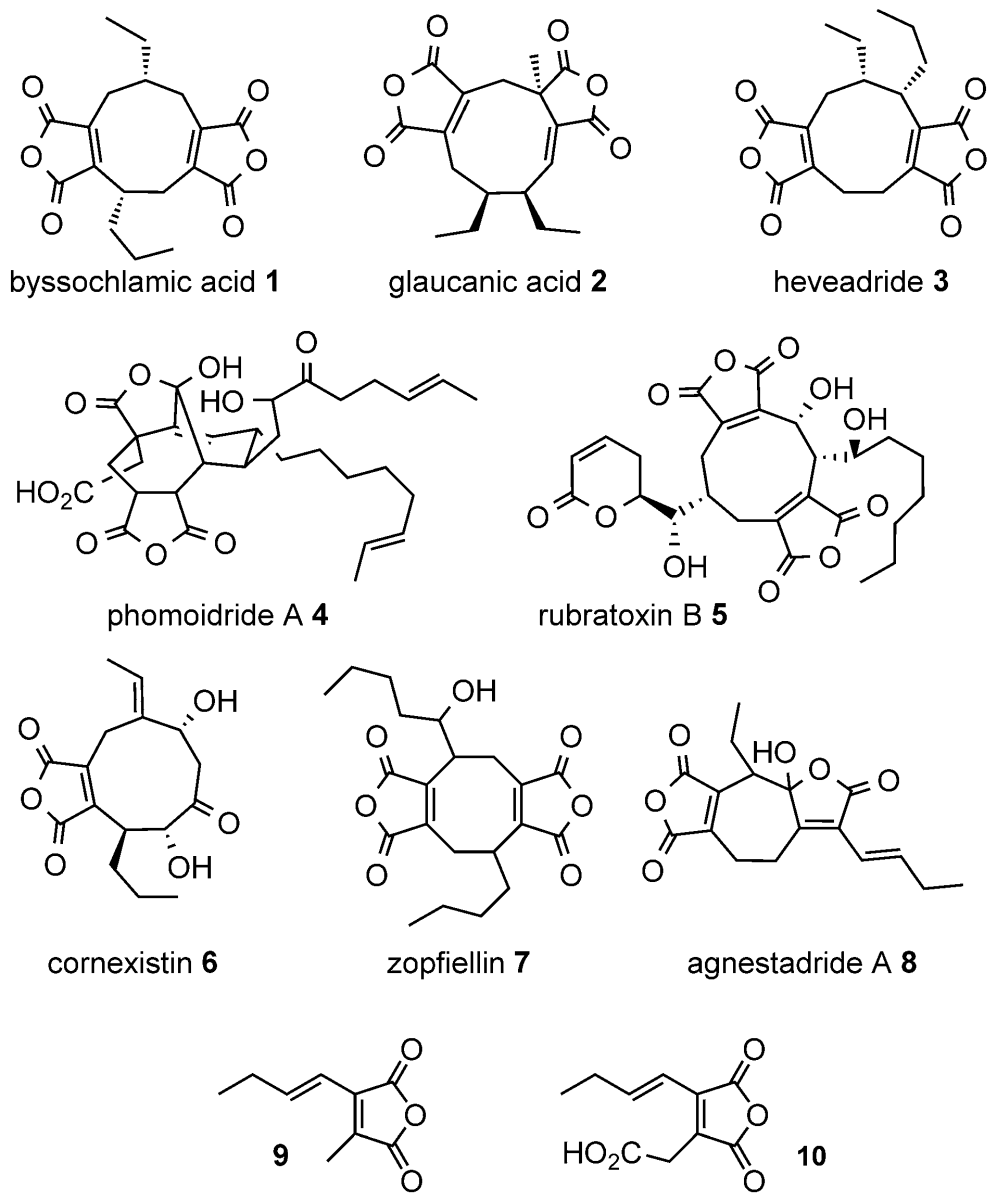
Examples of maleidrides and the hypothesized precursor.

In 1965, Barton and Sutherland proposed a scheme for the biosynthesis of **1**, **2**, and **3**.^3^ They envisaged biosynthesis from the C_9_‐maleic anhydride monomer **9**, with the differences in the position of the substituents explained by either “head‐to‐tail” or “head‐to‐head” dimerization. However the molecular basis for this dimerization has remained unknown until now.

A reinvestigation of the metabolites produced by *B. fulva* led to the identification of the probable monomer **10**, which undergoes facile decarboxylation to **9**. Decarboxylation is also proposed to produce the *exo*‐methylene putative intermediate **11** (Scheme 1),^10^ which can then undergo cyclization with a second molecule of **10** to produce **1**. Another mode of dimerization of **10** (head‐to‐side dimerization) explains the biosynthesis of agnestadride A (**8**).^10^

**Scheme 1:**
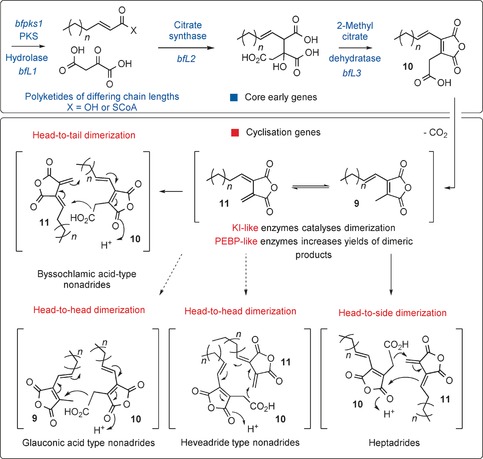
Proposed general pathway to maleidrides.

Barton and Sutherland also hypothesized that monomer **9** is a modified product of the citric acid cycle, produced from a polyketide‐derived hexanoate with oxaloacetate.^3^ Isotopic feeding studies supported this hypothesis^11^ and suggested that a candidate maleidride biosynthetic gene cluster (BGC) should contain a highly reducing polyketide synthase (hrPKS),^12^ as well as a gene encoding a citrate‐synthase‐like enzyme.^13^

Based on this hypothesis, we sequenced the genomes of two *B. fulva* strains, IMI 40021 and IMI 58422, both of which are linked to the original strain investigated by Raistrick and Smith.^1^ The former reliably produces byssochlamic acid, but the latter is an unreliable and generally poor producer. BLAST^14^ analyses were utilized to search the genomes of both *B. fulva* strains for likely maleidride BGCs, which led to the identification of a highly homologous maleidride‐type BGC in each genome (Figure 2, Genbank accession KU928136). Each BGC contains an hrPKS, a citrate‐synthase‐like enzyme, and several other interesting genes, including a methylcitrate dehydratase. Transcriptomic analysis of *B. fulva* IMI 40021 under byssochlamic acid producing and non‐producing conditions confirmed that the putative BGC is highly differentially expressed (Figure 2). The two BGCs are more than 97 % identical but show differences in the *bfR4* promoter region, which might explain the low productivity we observed in the IMI 58422 strain (see the Supporting Information).

**Figure 2:**
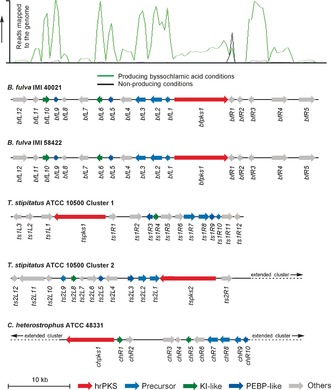
Top: transcriptome analysis of the byssochlamic acid (**1**) biosynthetic gene cluster in *B. fulva* IMI 40021 under producing (green) and non‐producing (black) conditions. Bottom: comparative genomic analysis of other putative maleidride BGCs.

BLAST^14^ searches of genome sequences deposited at the NCBI, utilizing *bfpks1* and the citrate synthase‐like gene (*bfL2*) as queries, revealed several further likely maleidride BGCs in different fungi. Two BGCs were identified from *Talaromyces stipitatus*. Many *Talaromyces* species are known to produce glaucanic acid (**2**), as well as the more complex rubratoxin B **(5**).^15^ A third BGC was identified in a *Cochliobolus* species. *Cochliobolus* species are synonymous with *Helminthosporium*, which are the original producers of heveadride (**3**).^5^, ^16^

The *bfpks1* gene that encodes the PKS was knocked out by using the bipartite method^17^ to produce *B. fulva bf*Δ*pks1* strains, which no longer produced byssochlamic acid (**1**) or agnestadride A (**8**; Figure 3 A and 3 B). This is consistent with heptadride **8** being formed from the same pathway as byssochlamic acid.

**Figure 3:**
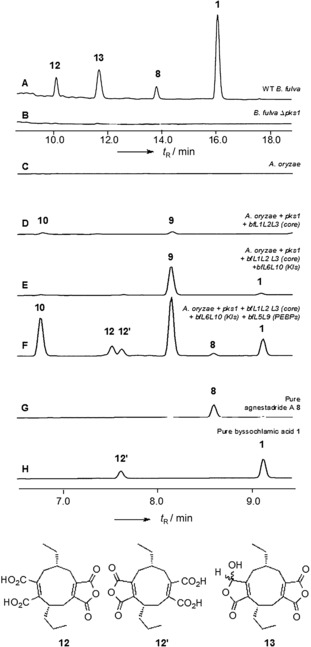
HPLC (DAD, 210–600 nm) chromatograms. A) Extract from WT *B. fulva*; B) extract from *B. fulva* Δ*pks1*; C) extract from untransformed *A. oryzae* NSAR1; D) extract from A. oryzae transformant BF‐PMCH; E) extract from *A. oryzae* transformant BF‐PMCH+KIs; F) extract from *A. oryzae* transformant BF‐PMCH+KIs+PEBPs; G) Purified agnestadride A (**8**); H) Purified byssochlamic acid (**1**). Compounds **12**, **12′**, and **13** are cometabolites of **1** and have been previously identified and characterized.^10^

In a recent investigation of the biosynthesis of phomoidride A (**4**), Oikawa and co‐workers also identified a BGC containing an hrPKS, a citrate‐synthase‐like enzyme, and a methylcitrate dehydratase, as well as the two maleidride‐type BGCs from *Talaromyces stipitatus*.^18^ Heterologous expression experiments utilizing the three genes from one of the *T. stipitatus* BGCs allowed the isolation of a longer‐chain decarboxylated monomer. These experiments confirm the polyketide/oxaloacetate origin of maleidride monomers.^18^ However the proteins responsible for the key cyclisation reaction necessary for maleidride biosynthesis remain unkown.

From comparison of the two *B. fulva* BGCs and the other BGCs identified above, four highly conserved genes were chosen for heterologous expression experiments in *Aspergillus oryzae* NSAR1:^19^, ^20^ the hrPKS *bfpks1*; the citrate‐synthase‐like gene *bfL2*; the methylcitrate dehydratase *bfL3*; and the hydrolase 341 *bfL1*, which encodes a protein homologous to Type II thiolesterases such as RifR,^21^ which are involved in the release of abberant ACP‐bound polyketide intermediates during modular polyketide biosynthesis, as well as to LovG, which releases the lovastatin nonaketide from its PKS.^22^

Expression of these four core genes in *A. oryzae* NSAR1 (strains AO‐BF‐PMCH 1–7) led to the production of both **9** and **10** (Figure 3 D). Compound **9** is already known to be the decarboxylation product of the highly volatile and unstable **10**.^10^ We also expressed *bfpks1*, *bfL2*, and *bfL3* in the absence of *bfL1*, which encodes the hydrolase. Under these conditions, **9** and **10** were not produced (see the Supporting Information), thus strongly suggesting that the hydrolase is essential in the byssochlamic acid system.

Oikawa and co‐workers suggested that the longer‐chain decarboxylated monomer isolated from their heterologous expression experiments is produced by an adventitious native decarboxylase.^18^ Our experiments have previously identified the natural product **10** by comparing its LCMS characteristics with data collected for the synthetic homologue, which was also observed to decarboxylate spontaneously to give **9**. This demonstrates that, at least for the shorter‐chain maleic anhydrides, no enzymatic decarboxylation is necessary.^10^

To investigate the dimerization process, several further genes were selected for heterologous expression. Analysis of the BGC comparisons from several organisms (Figure 2) revealed that in addition to the core genes, two further types of genes are common to each BGC, namely either one or two genes encoding putative ketosteroid isomerase (KI)‐like proteins and either one or two putative phosphatidylethanolamine‐binding proteins (PEBP).

Co‐expression of the two KI‐like genes (*bfL6* and *bfL10*) with the core genes (*bfpks1*, *bfL1*, *bfL2*, and *bfL3*) in the heterologous host (strains AO‐BF‐PMCH+KIs 1–21) led to the production of byssochlamic acid (**1**) and the decarboxylated intermediate **9** in several transformants (Figure 3 E). Agnestadride A (**8**) and the intermediate **10** were also detected, but in low titer (see the Supporting Information). This result shows that in the *A. oryzae* NSAR1 background, the four core “monomer” genes and the KI‐like genes are sufficient to form both nonadrides and heptadrides.

To determine whether the presence of both KI‐like genes is necessary for dimerization, or alternatively, whether each KI‐like gene controls a different dimerization mode (nonadride/ heptadride), the core “monomer” genes were co‐expressed with either KI1 (*bfL6*) or KI2 (*bfL10*) alone to give strains AO‐BF‐PMCH+KI1 1–7 and AO‐BF‐PMCH+KI2 1–7. Both sets of strains only produced **9** and **10**, with no evidence for dimerized products (see the Supporting Information). Reverse‐transcription PCR (RT‐PCR) confirmed that both KI1 and KI2 genes were expressed in each experiment, therefore it can be concluded that within the *A. oryzae* NSAR1 background, the core “monomer” genes and KI1 or KI2 alone are not sufficient to catalyze dimerization.

The BGC comparisons also revealed that genes encoding proteins similar to PEBPs are common to all of the maleidride BGCs (Figure 2). The two PEBP genes (*bfL5* and *bfL9*) were thus co‐expressed with the core genes and the KI‐like genes in *A. oryzae* NSAR1 to create strains AO‐BF‐PMCH+KIs+PEBPs 1–8. No new compounds were identified from extracts from these strains. However, direct quantitative comparisons between strains containing the PEBP genes in addition to KI genes showed an over 20‐fold increase in the biosynthesis of byssochlamic acid (to 44 mg L^−1^, Figure 3 F), as well as a corresponding increase in the production of the heptadride **8**. Byssochlamic acid (**1**) isolated from these experiments was chromatographically and spectroscopically identical to **1** isolated from wild‐type (WT) *B. fulva* (see the Supporting Information). Compounds **12** and **12′** were also observed in these experiments: these are most likely formed in vitro through partial hydrolysis of **1** during extraction and analysis. However, the reduction product **13** is not formed in *A. oryzae*, thus suggesting that **13** arises in *B. fulva* through the activity of an adventitious enzyme.

The biosynthetic pathway to byssochlamic acid and agnestadride A has thus been fully elucidated through genome and transcriptome sequencing, gene disruption, and heterologous expression. Putative maleidride BGCs were identified from the genomes of *Talaromyces* and *Cochliobolus* species. Both genera are known to produce nonadrides. Comparison of the byssochlamic acid cluster to the putative maleidride gene clusters identified a core set of genes that are common to all five BGCs. Four genes were identified (*bfpks1*, *bfL1*, *bfL2*, and *bfL3*) that, when expressed in the heterologous host *A. oryzae* NSAR1, produce the monomer **10**, as well as its spontaneous decarboxylation product **9**, both of which have been previously identified from WT *B. fulva* extracts.^10^ Experiments to determine the genes involved in dimerization demonstrated that both KI‐like genes co‐expressed with the core genes are necessary. Furthermore, co‐expression of the core genes with both KI‐like genes and both PEBPs gave increased yields of dimerized products. The mechanism for the dimerization still needs to be established through in vitro experiments.

Based on previous biosynthetic investigations, our discovery of the byssochlamic acid pathway, and research conducted by Oikawa and co‐workers,^18^ we now propose a general biosynthetic route to maleidrides (Scheme 1). In each maleidride pathway, the initial steps are common, with potential differences including polyketide chain length and pattern of reduction. For example, the biosynthesis of **1**–**3**, **6**, and **8** requires triketides; **5** requires pentaketides; and **4** requires hexaketides. The polyketide is presumably released from the PKS by the BfL1 hydrolase, although it is not yet known whether this is as the free acid or as a CoA‐thiolester. This result contrasts with the recently reported work of Oikawa, who showed that the hydrolase did not appear to be required in the case of a Talaromyces PKS.^18^ However it is known that some iterative fungal PKSs, for example the squalestatin tetraketide synthase,^23^ can release polyketides without the requirement for a dedicated thiolesterase. Reaction between the polyketide and oxaloacetate, catalyzed by the citrate‐synthase‐like enzyme, is followed by dehydration by the methylcitrate dehydratase homologue, which then sets up the synthesis of the maleic anhydride decarboxylated monomers **9** and **11**. It is interesting to note that fungi have evolved at least two distinct routes to maleic anhydride moieties. Other organisms oxidize vicinal aromatic methyl groups to form this biologically important motif.^24^, ^25^

The KI‐like enzyme(s) can then react **10** with either **9** or **11**. These differences, and the orientation of the reacting species, determine the size, substitution pattern, and stereochemistry of the central carbocyclic ring. Previous in vitro work by Baldwin and others^26^ has shown that such reactions are chemically feasible, although in the absence of enzymes, strong bases are required and low yields are observed. Our experiments suggest that the KI‐like proteins from *B. fulva* act together to form heterodimeric enzymes that show “flexibility” in that they create both nonadrides and heptadrides. To our knowledge, *B. fulva* IMI 40021 is the first fungus known to produce cross‐class maleidrides (i.e., nonadride and heptadride), so this “flexibility” may be specific to the byssochlamic acid/agnestadride A pathway. Other known maleidride producers appear to have pathways that only involve one class of dimerization, although it is possible that reinvestigation of the metabolites produced may identify other minor compounds with different dimerization modes. The PEBP‐like enzymes also appear to be involved in the dimerization, and although their catalytic role is not yet clear, it is possible their known anionic binding ability may be involved.^27^ One possibility may be the chaperoning of highly unstable carboxylates such as **10** in order to prevent premature decarboxylation prior to dimerization. However further understanding of the role of the KI and PEBP proteins must await in vitro experiments. Gene clusters encoding the biosynthesis of octadrides such as zopfiellin (**7**) have not yet been reported, but our results suggest that similar KI‐like and PEBP‐like proteins may be involved in their biosynthesis.

In conclusion, our experiments have revealed for the first time that maleidride biosynthesis can be recreated in a heterologous host and the observations open the way for future experiments to create new compounds in this class with potentially interesting and useful biological properties through pathway engineering. Further experiments are currently underway in our laboratories to understand the mechanisms and selectivity of the ring‐forming enzymes.

## Supporting information

As a service to our authors and readers, this journal provides supporting information supplied by the authors. Such materials are peer reviewed and may be re‐organized for online delivery, but are not copy‐edited or typeset. Technical support issues arising from supporting information (other than missing files) should be addressed to the authors.

Supplementary
