# Supplementary material for: Heterologous Production of Fungal Maleidrides Reveals the Cryptic Cyclization Involved in their Biosynthesis
Source: Angew Chem Int Ed Engl. 2016 Apr 21;55(23):6784–8. doi: 10.1002/anie.201511882 (PMC4982102; doi:10.1002/anie.201511882)
Supplement: Supplementary file 1 — Supplementary [file ANIE-55-6784-s001.pdf]

Angewandte Chemie
Eine Zeitschrift der Gesellschaft Deutscher Chemiker

Supporting Information

Heterologous Production of Fungal Maleidrides Reveals the Cryptic Cyclization Involved in their Biosynthesis

Katherine Williams, Agnieszka J. Szwalbe, Nicholas P. Mulholland, Jason L. Vincent, Andrew M. Bailey, Christine L. Willis, Thomas J. Simpson, and Russell J. Cox**

anie_201511882_sm_miscellaneous_information.pdf

Katherine Williams,[a]* Agnieszka J. Szwalbe,[b] Nicholas P. Mulholland,[c] Jason L. Vincent,[c] Andrew M. Bailey,[d] Christine L. Willis,[b] Thomas J. Simpson[b] and Russell J. Cox[a,b]*

a. Institute for Organic Chemistry, Leibniz University of Hannover, Schneiderberg 1B, 30167, Germany
b. School of Chemistry, University of Bristol, Cantock's Close, Bristol, BS8 1TS, UK.
c. Syngenta, Jealott's Hill, Bracknell, Berkshire, RG42 6EY, UK.
d. School of Biological Sciences, Bristol Life Sciences Building, University of Bristol, 24 Tyndall Ave, Bristol BS8 1TH, UK.

*corresponding authors: russell.cox@oci.uni-hannover.de;
katherine.williams@oci.uni-hannover.de

Electronic Supplementary Information

Contents

1. General procedures 3
2. Strains 3
3. Growth and fermentation conditions 3
4. Fungal nucleic acid preparation 3
5. Genome and RNA sequencing 3
6. LCMS analysis 3
7. Analysis of byssochlamic acid cluster from *B. fulva* IMI 40021 4
   7.1 Bioinformatic analysis of 'core' genes from *B. fulva* IMI 40021 5
      7.1.1 PKS 5
      7.1.2 Citrate synthase-like gene 5
      7.1.3 2-methylcitrate dehydratase 7
      7.1.4 Hydrolase 341 8
      7.1.5 KI-like genes and PEBPs 9
      7.1.6 Promoter region of *bfR4* 10
8. Comparison of maleidride-type clusters 11
   8.1 Artemis comparison 11
   8.2 Homology comparison tables 12
9. Primer table 13
10. Plasmid construction 14
   10.1 pE-YA-bfpks1 14
   10.2 pTYarg-BF-PMCH 14
   10.3 pTYade-GS-KIs 15
   10.4 pTYsC-GS-PEBPs 15
   10.5 pTYade-GS-KI1 and pTYade-GS-KI2 15
11. Transformation 16
   11.1 *S. cerevisiae* 16
   11.2 *A. oryzae* 16

11.3 *B. fulva* IMI40021 16
12. *bfpks1* gene disruption 17
12.1 Genetic characterization of gene disruption 17
13. Metabolite extraction and LCMS analysis 18
13.1 *B. fulva* IMI 40021 18
13.2 LCMS analysis of standards 18
13.3 LCMS analysis of *B. fulva* strains 19
13.4 Metabolite extraction and LCMS analysis of *A. oryzae* NSAR1 19
13.5 LCMS analysis of *A. oryzae* NSAR1 transformants 20
13.5.1 Comparison between *A. oryzae* strains 20
13.5.2 LCMS analysis of strain AO-BF-PMCH 21
13.5.3 LCMS analysis of strain AO-BF-PMCH+KIs 22
13.5.4 LCMS analysis of AO-BF-PMCH+KI1/2 strains 23
13.5.5 LCMS analysis of strain AO-BF-PMCH+KIs+PEBPs 24
13.5.6 Direct LCMS comparison of strains AO-BF-PMCH+KIs and AO-BF-PMCH+KIs+PEBPs 25
14. RT-PCR analysis of strains AO-BF-PMCH+KI1 1-7 and AO-BF-PMCH+KI2 1-7 26
15. Coexpression of *bfpks1*, *bfL2* and *bfL3* 26
16. Analysis of purified byssochlamic acid **1** from expression experiments 27
17. Stereochemistry of byssochlamic acid **1** from *A. oryzae* AO-BF-PMCH+KIs+PEBPs 29
18. References 30

1. General procedures

Analytical grade chemicals and reagents were supplied from Sigma-Aldrich, Alfa Aesar, Acros Organics, Becton-Dickinson, BDH, Fischer, Fluka and Difco, unless otherwise stated. Solvents used for LC-DAD-MS analyses were HPLC grade. General molecular biology procedures were performed as standard[1] and molecular biology kits used according to manufacturer's protocols. Analytical PCR was performed using BioMix Red (Bioline) and preparative PCR was performed using Phusion polymerase (NEB).

2. Strains

Escherichia coli strain TOP10 (Invitrogen) was used as a host for standard plasmids. Gateway destination vectors were propagated in *ccdB*-Survival cells (Invitrogen). *Saccharomyces cerevisiae* strain YPH499 (Stratagene) was used a host for plasmid assembly by homologous recombination. *A. oryzae* NSAR1 was obtained as a gift from the Kitamoto group.[2] *B. fulva* IMI 40021 and IM 58422 were purchased from CABI.[3]

3. Growth and fermentation conditions

A. oryzae NSAR1 was maintained on MEA, liquid induction medium was 100 ml CMP in 500 ml flasks (3.5% *w/v* Czapek Dox Broth, 2% *w/v* D-(+)-maltose monohydrate, 1% *w/v* peptone) at 28 °C with shaking at 200 rpm for 7 days. *B. fulva* strains IMI 40021 and IMI 58422 were maintained on PDA, liquid induction medium was 100 ml CGB in 500 ml flasks (5% *v/v* of solution A (4% *w/v* $NaNO_3$, 1% *w/v* KCL, 1% *w/v* $MgSO_4.7H_2O$, 0.02% *w/v* $FeSO_4.7H_2O$), 5% *v/v* of solution B (2% *w/v* K_2HPO_4), 0.1% *v/v* of solution C (1% *w/v* $ZnSO_4.7H_2O$), 0.1% *v/v* of solution D (0.5% *w/v* $CuSO_4{\cdot}5H_2O$) and 3% *w/v* of D-(+)-glucose,) with static culturing at 25 °C, for 12 days.

4. Fungal nucleic acid preparation

Fungi were cultured in the appropriate liquid medium, which was then pelleted, lyophilized and ground under liquid nitrogen. Genomic DNA for both sequencing and PCR analysis was prepared using the GenElute Plant Genomic DNA Miniprep kit (Sigma). RNA was prepared using the RNeasy Plant Mini Kit (Qiagen) for both sequencing and RT-PCR analysis. RNA was reverse transcribed using the First Strand cDNA synthesis Kit (Thermo Scientific).

5. Genome and RNA sequencing

Sequencing of an Illumina mate-pair genomic DNA library for *B. fulva* strain IMI 58422 was conducted using paired-end (100bp reads) sequencing on the Illumina HiSeq 2000 platform. Over 30 Mb were sequenced and assembled into scaffolds with an N50 of over 4 Mb. Genomic DNA of *B. fulva* strain IMI 40021 was subjected to paired-end Illumina sequencing to give over 31 Mb after assembly with an N50 of over 287 kb. RNA was extracted from *B. fulva* IMI 40021 from both producing (static fermentation in CGB medium at 25 °C for 12 days) and non-producing (shaking fermentation at 200 rpm in CGB medium at 25 °C for 12 days) conditions. Paired-end Illumina sequencing was conducted after TruSeq RNAseq library preparation. The sequencing reads were mapped to the genome assembly using Tophat.[4]

6. LCMS analysis

LC-MS data were obtained using a Waters LCMS system comprising of a Waters 2767 autosampler, Waters 2545 pump system, a Phenomenex Kinetex column (2.6 μ, C_{18}, 100 Å, 4.6 × 100 mm) equipped with a Phenomenex Security Guard precolumn (Luna C_5 300 Å) eluted at 1 mL/min. Detection was by Waters 2998 Diode Array detector between 200 and 600 nm; Waters 2424 ELSD and Waters SQD-2 mass detector operating simultaneously in ES+ and ES- modes between 100 *m/z* and 800 *m/z*. Solvents were: A, HPLC grade H_2O containing 0.05% formic acid; and B, HPLC grade CH_3CN containing 0.045% formic acid. Gradients were as follows. *Method 1* (used for all *A. oryzae* extractions). Kinetex/CH_3CN: 0 min, 10% B; 10 min, 90% B; 12 min, 90% B; 13 min, 10% B; 15 min, 10% C. *Method 2* (used for all *B. fulva* extractions). Kinetex/CH_3CN 0 min. -5% B, 2 min. -40% B, 20 min. -90% B, 22 min. -95% B, 26-30 min. – 5% B.

7. Analysis of byssochlamic acid cluster from *B. fulva* IMI 40021

Softberry FGENESH[5] was utilized for predictions of the intron and exon positions for coding sequences within the BGC, which were manually adjusted by comparison with the transcriptomics data. Coding sequences were then annotated using BLAST[6] and InterPro (Table S1).[7]

Table S1: Analysis of the putative byssochlamic acid and agnestadride A BGC from *B. fulva* IMI40021 (Accession number: KU928136). *Closest characterized homologue in the Swissprot database

BF Gene	Putative function	Homologue*	Identity (%)	E value	Query coverage	Score
bfpks1 **(ANF07288)**	Polyketide synthase	LDKS[8]	33%	0	98%	1320
bfL1 **(ANF07287)**	Hydrolase 341	FSH2[9]	30%	2e-07	59%	53.1
bfL2 **(ANF07286)**	Citrate synthase-like	GltA[10]	33%	2e-74	96%	248
bfL3 **(ANF07285)**	2-methyl citrate dehydratase	PDH1[11]	42%	1e-130	97%	395
bfL4 **(ANF07284)**	MFS transporter	Qdr2p[12]	31%	8e-67	100%	230
bfL5 **(ANF07283)**	Phosphatidylethanolamine-binding protein (PEBP)	hPEBPP4[13]	28%	1e-05	53%	47.8
bfL6 **(ANF07282)**	Ketosteroid isomerase-like	-	-	-	-	-
bfL7 **(ANF07281)**	C6 transcription factor	aflR[14]	29%	1e-07	14%	57
bfL8 **(ANF07280)**	Regulatory protein	-	-	-	-	-
bfL9 **(ANF07279)**	Phosphatidylethanolamine-binding protein (PEBP)	-	-	-	-	-
bfL10 **(ANF07278)**	Ketosteroid isomerase-like	-	-	-	-	-
bfL11 **(ANF07277)**	Enoyl-CoA hydratase/isomerase family protein	ECH1[15]	28%	7e-15	96%	75.5
bfL12 **(ANF07276)**	MFS transporter	mirB[16]	38%	2e-119	96%	374
bfR1 **(ANF07289)**	DUF 636	-	-	-	-	-
bfR2 **(ANF07290)**	Sodium/solute transporter	DUR3[17]	45%	0	100%	740
bfR3 **(ANF07291)**	Seed maturation protein	-	-	-	-	-
bfR4 **(ANF07292)**	C6 transcription factor	LEU3[18]	22%	2e-25	85%	115
bfR5 **(ANF07293)**	Small oligopeptide transporter	isp4[19]	45%	0	100%	740

7.1 Bioinformatic analysis of 'core' genes from *B. fulva* IMI 40021

7.1.1 PKS

Analysis of the putative byssochlamic acid hrPKS (*bfpks1*) using InterPro[7] showed that it contains all of the domains normally present in a fungal hrPKS: a ketosynthase (KS - IPR020841); an acyltransferase (AT - IPR020801); a dehydratase (DH - IPR020807); a *C*-methyltransferase (*C*Met - IPR029063); an enoyl reductase (ER - IPR020843); a ketoreductase (KR - IPR013968); and an acyl-carrier protein (ACP - IPR020806).[20] Corresponding conserved motifs for each domain were apparent except for the *C*Met domain.[21, 22, 23, 24, 25] Motif I in the *C*Met domain has a mutation in the conserved Glycine residue (underlined - [LIV][VL][ED][VI]<u>G</u>xGxG), but since there is no expected methylation in the polyketide chain of byssochlamic acid, this is unsurprising.[26]

7.1.2 Citrate synthase-like gene

An alignment of the citrate synthase-like amino acid sequence (BFL2) with the citrate synthases from *Sus scrofa* and *Gallus gallus*[27] showed that it contains the conserved residues required for the active site (H 284, H 323 and D 377). Histidine-323 sits within the prosite[24] motif PS00480 (GX[GA]HX[<u>VI</u>]X[RTKQ]XX[DV][PS]R), with one mismatched residue (underlined) (Figure S1).

```
Clustal Muscle

BFL2                     MSSGTLFVKDSRTSLNYEIPIHRNAIAATAFKKIKA-PVSGSDPADKVDGGLRVHDPGLQ
4CTS_A                   ASSTNL--KDILADL-----IPKEQARIKTFRQQHGNTVVGQITVDMMYGGMRGMKGLVY
6CSC_A                   ASSTNL--KDVLASL-----IPKEQARIKTFRQQHGNTAVGQITVDMSYGGMRGMKGLIY
                          ** .*  **  :.*     * .:    .:*.: :. .. *. ..*   **:*  .  :

BFL2                     NTTVVETDISFSNSDSGLLLFRGYSLDQLW----------DSDFEELFHLLVWGKYPTRV
4CTS_A                   ETSVLDPDEG--------IRFRGYSIPECQKMLPKAKGGEEPLPEGLFWLLVTGQIPTEE
6CSC_A                   ETSVLDPDEG--------IRFRGFSIPECQKLLPKAGGGEEPLPEGLFWLLVTGQIPTPE
                         :*:*::.* .        : ***:*: : .          :.  * **.*** *: **

BFL2                     QKDDLSNTLANYMKNVPENVVKAIRSFPRSTSPMPMVIAGLAAYIASDPESIPAANGG--
4CTS_A                   QVSWLSKEWAK-RAALPSHVVTMLDNFPTNLHPMSQLSAAITA-LNSESNFARAYAEGIH
6CSC_A                   QVSWVSKEWAK-RAALPSHVVTMLDNFPTNLHPMSQLSAAITA-LNSESNFARAYAEGIN
                         * . :*:  *:    :*.:**. : .** .  **. : *.::* : *:.:   *   *

BFL2                     -NIYQGNTAQTDLGILKTVSAYAVVLGLVASHRKDIPFVPASSENSYYENLFIMMGMVDR
4CTS_A                   RTKYWELIYEDCMDLIAKLPCVAAKI-YRNLYREGSSIGAIDSKLDWSHNFTNMLGYTD-
6CSC_A                   RTKYWEFVYEDAMDLIAKLPCVAAKI-YRNLYRAGSSIGAIDSKLDWSHNFTNMLGYTD-
                          . *.    :  :.:: .:.. *. :     :* . .: . .*: .:  *:  *:* .*
                                                                                  H 284
BFL2                     VSGRPDTLQLSCFRRFAALSCDN-GMALSVFATLVCASSLADPISCLISALAAAYGPLHF
4CTS_A                   ----AQFTEL--MRLYLTIHSDHEGGNVSAHTSHLVGSALSDPYLSFAAAMNGLAGPLHG
6CSC_A                   ----PQFTEL--MRLYLTIHSDHEGGNVSAHTSHLVGSALSDPYLSFAAAMNGLAGPLHG
                             .:  :*  :* : :: .*: *  :*..:: : .*:*:**  .: :*: .  ****
                                                          H 323
BFL2                     GATEAA----HRALQEIG---SVERVPDFLEQVKRGERKLFGYGHRTYKGTDPRVIPIKK
4CTS_A                   LANQEVLVWLTQLQKEVGKDVSDEKLRDYIWNTLNSGRVVPGYGHAVLRKTDPR-YTCQR
6CSC_A                   LANQEVLLWLSQLQKDLGADASDEKLRDYIWNTLNSGRVVPGYGHAVLRKTDPR-YTCQR
                          *.: .     .  :::*   * *.: *:: :. .. * : **** . . ****  . :.
                                                         D 377
BFL2                     LLEDSNATSNPLIEIAKSIEIHASTDDYFKSRGLS----ANADFYGNFVFSAIGF-DPDF
4CTS_A                   EFALKHLPHDPMFKLVA--QLYKIVPNVLLEQGKAKNPWPNVDAHSGVLLQYYGMTEMNY
6CSC_A                   EFALKHLPSDPMFKLVA--QLYKIVPNVLLEQGKAKNPWPNVDAHSGVLLQYYGMTEMNY
                          :  .: . :*::::.   :::  . : : ..* :    .*.* :...::.  *: : ::

BFL2                     IPVAMLAQRIIGIMAH--WREYMLKRGKLFRPSHIYTGNTEPLCNFSPKL
4CTS_A                   YTVLFGVSRALGVLAQLIWSRAL--GFPLERPKSMSTDGLIKLVDSK---
6CSC_A                   YTVLFGVSRALGVLAQLIWSRAL--GFPLERPKSMSTAGLEKLSAGG---
                          .* : ..* :*::*:  *   :     * **. : * .   *
```

Figure S1: Alignment of BFL2 (citrate synthase) from the byssochlamic acid cluster with the citrate synthases from *Sus scrofa* (4CTS_A) and *Gallus gallus* (6CSC_A). Conserved residues from the active site are highlighted in red. The prosite motif PS00480 (GX[GA]HX[<u>VI</u>]X[RTKQ]XX[DV][PS]R) is highlighted by the red box. One residue (underlined) is not conserved in BFL2.

Subcellular localization prediction (TargetP)[28] showed that BFL2 was not likely to be targeted to the mitochondrion, unlike the test set of known citrate synthases examined (Table S2). TS1R7 and TS2L2 (the citrate synthases from the putative *Talaromyces* maleidride BGCs) were also not expected to be targeted to the mitochondrion, however the CHR8 (the citrate synthase from the putative *Cochliobolus* maleidride BGC) prediction shows a weak match for mitochondrial targeting (Table S2).

Table S2: Predictions of subcellular localization based on N-terminal amino acid sequences of various citrate synthase genes. Neural network scores are shown, as well as a reliability class for each prediction (1 being most reliable, 5 being least). Location M denotes prediction of localization to the mitochondrion.

Name	Length	Mitochondrial targeting peptide	Signal peptide (Secretory)	Other	Location	Reliability Class
XP_681544.1[29]	474	0.923	0.019	0.103	M	1
XP_002485078.1	473	0.925	0.016	0.119	M	1
XP_014080474.1	467	0.949	0.025	0.074	M	1
XP_002479653.1	467	0.775	0.041	0.195	M	3
XP_014084782.1	471	0.927	0.023	0.108	M	1
XP_664254.1[30]	460	0.907	0.028	0.138	M	2
BFCitSyn2	466	0.888	0.025	0.165	M	2
BFCitSyn1	474	0.916	0.014	0.128	M	2
P34085.2[31]	469	0.95	0.019	0.078	M	1
Q50I20.1[32]	465	0.935	0.025	0.088	M	1
TS1R7	438	0.116	0.071	0.892	_	2
TS2L2	443	0.107	0.046	0.917	_	1
CHR8	443	0.628	0.049	0.389	M	4
BFL2	441	0.298	0.048	0.754	_	3

Additionally, phylogenetic analysis of the citrate synthase-like genes identified from *B. fulva* and the other putative maleidride clusters showed that they cluster on a separate clade from the primary metabolism citrate synthases (Figure S2).

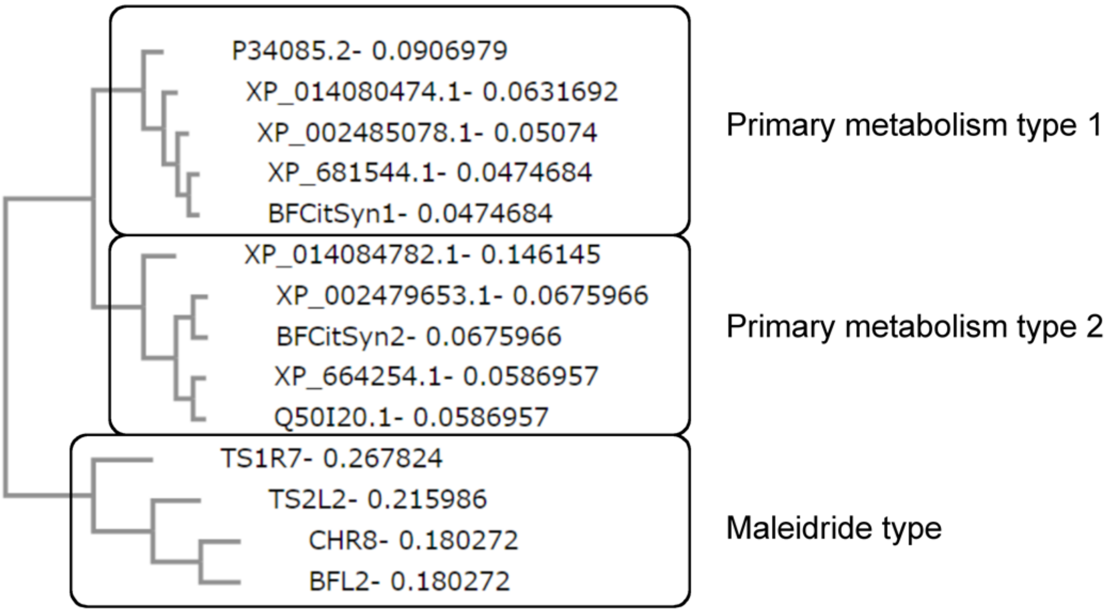

Figure S2: Phylogenetic analysis (utilizing UPGMA method) comparing known primary metabolism citrate synthases to the citrate synthases from *B. fulva* IMI 40021, *T. stipitatus* and *C. heterostrophus*. XP_681544.1 is a type 1 citrate synthase from *Aspergillus nidulans*.[29] XP_664254.1 is a type 2 citrate synthase also from *A. nidulans*.[30] P34085.2 is a type 1 citrate synthase from *Neurospora crassa*.[31] Q50I20.1 is a type 2 citrate synthase from *Aspergillus fumigatus*.[32] BFCitSyn1, XP_002485078.1 and XP_014080474.1 are putative type 1 citrate synthases from *B. fulva*, *T. stipitatus* and *C. heterostrophus* respectively. BFCitSyn2, XP_002479653.1 and XP_014084782.1 are putative type 2 citrate synthases from *B. fulva*, *T. stipitatus* and *C. heterostrophus* respectively.

7.1.3 2-methylcitrate dehydratase

2-Methylcitrate dehydratase enzymes are normally involved in the dehydration of 2-methylcitric acid to 2-methyl-*cis*-aconitic acid.[33] The sequence of BFL3 matches the InterPro motif for MmgE/PrpD family, which includes the 2-methylcitrate dehydratase (IPR012705).[7] A model of the protein structure of BFL3 aligns well to the crystal structure of a putative *Escherichia coli* 2-methylcitrate dehydratase (1szqa).[34] The model consists of an all α-helical domain linked to an α + β domain, with the active site likely to be positioned between the two domains.[35, 36]

```
CLUSTAL O(1.2.1) multiple sequence alignment


BFL3            ---------------------------------------------MSTEKYDSVIVDITEYVF
1SZQ            ------------------------------------MSAQINNIRPEFDREIVDIVDYVM
2HP0            -----------------MGSS---------HHHHHHSSGLVPRGSHMFTTKLAEKVVSAW
Q6C354.1        MRAFRSAANFGAASNIYRKSFTPASIASNRFVSARMSSIMTDNARPNTDKVVQDIADYIH
                                                   : : .

BFL3            RYRIESEKAWENARIALSDAVGCAIETVWKSESCRKLIGPAIPRTFTFDGFRLPGTAYQT
1SZQ            NYEISSKVAYDTAHYCLLDTLGCGLEAL-EYPACKKLLGPIVPGTVVPNGVRVPGTQFQL
2HP0            KAKI-SQPALKAAQDGVIDTVAAALGGVTEHSVQVAL--KYVAATGGSGDSKLWGVNQRS
Q6C354.1        DYKIDSSVAMETARLCFLDTLGCGLEGL-KYQQCANIVGPVVPGTIVPNGTKVPGTDYQV
                  .* *. * . *:  . *::...:  : :      :    :  *      :: *.  :

BFL3            DPIKGAFDMATIIRYLDHNDAIAGADWGHPSDNIG-AILAVMDWLSRSVESGKLVHTGPP
1SZQ            DPVQAAFNIGAMIRWLDFNDTWLAAEWGHPSDNLG-GILATADWLSRNAVAS---GK-AP
2HP0            NMFDAAFVNGMAAHAIDFDDSFPV-MRGHPSSSLVPAIFAVGEHVGANGHNC---LKSYV
Q6C354.1        DPVRGAFNIGTIIRWLDFNDCWLAAEWGHPSDNLG-GILAVADWQTRSAKAG---LEGKV
                : . .**  .   : :*.:*       ****..:  .*:*. :    .

BFL3            LTIRTL---LTAIIKAYEIQGCFLLHNAFNSHGLDHVILVKLASTALVCWLLGLTETHTM
1SZQ            LTMKQV---LTAMIKAHEIQGCIALENSFNRVGLDHVLLVKVASTAVVAEMLGLTREEIL
2HP0            LGIEVVATLGRAVGKGHYLAGWHP-----------TSTLGVFGATTAAALLLGADEEQLR
Q6C354.1        FKVKDV---LEGMIKAHEIQGGLAIENSFNRVGLDHVVLVKIASTAVVSGMLGLSREQTA
                : :. :     .: *.: : *                 *  ..:*: .. :**  . .

BFL3            AAISHVWMDGHP-LRVYRSGSNTTPRKGWAAGEACMKAVQLAFLVREGQPGSPTVLTM--
1SZQ            NAVSLAWVDGQS-LRTYRHAPNTGTRKSWAAGDATSRAVRLALMAKTGEMGYPSALTA--
2HP0            NAWGIAASNSCGIIKNFGT-----MTKPMHTGSAARNGVLSAWLSMQSFTGCQTVFDDAE
Q6C354.1        DAISQAFVDGQS-LRTYRHAPNTMSRKSWAAGDATSRAVNLALLVKKGEGGMPSILTA--
                 * . .  :.   :: :         *   :*.*  ..*  * :   .  *  : :

BFL3            PRWGFYATMFGGKEFTFPINYST-W-VIEHVFFKV-MPVEGHGVAAVEAALIIHKILRQH
1SZQ            PVWGFYDVSFKGESFRFQRPYGS-Y-VMENVLFKISFPAEFHSQTAVEAAMTLYEQMQAA
2HP0            GILAMYGAQPGPELFNAMQKFGTPWAIIAPGLYKKSWPSCYANHKPLA---GLFAIMKEH
Q6C354.1        KTWGFYDVLFGGKEFKFQRPYGS-Y-VMENVLFKISFPAEFHAQTACESAMLLHEELKKL
                   .:* .    : *     :.: : ::   ::*   *              :.  ::

BFL3            SLSADKDIKSIEIRTNAAANMIINKSGRLRNSADRDHCMQYLVALAFLKGEVPEPEDFLD
1SZQ            GK-TAADIEKVTIRTHEACIRIIDKKGPLNNPADRDHCIQYMVAIPLLFGRLTAA-DYED
2HP0            GL-TGQDISHVDVGFLPGVEKPLLYMDP-RTTEEAKFSIEANIGAALLDGEVSLA-SFEI
Q6C354.1        GK-TSDDIASIKIRTQEAAMRIIDKKGPLHNYADRDHCIQYMVAIPLIHGRLTAD-DYTD
                .  :  **  : :    .    :      ..  : ...::  :.  :: *.:    .:

BFL3            NSPWANDSVLNILRDRILIRTDDQLTHDYMDLGKKSLACGVTVHLINGEVIDEVLVEFPV
1SZQ            --NVAQDKRIDALREKINCFEDPAFTADYHDPEKRAIANAITLEFTDGTRFEEVVVEYPI
2HP0            --EHLDRPAMRAAMKKVTRF---------D--------MPSETTFSGTTGYTDIVVHTAD
Q6C354.1        --EIASDPRIDALREKMECVEDKRFSEEYHAPDKRYIGNAIEITLKDGTVLDEIEVNYPI
                     .   :    .::                           :       :: *.

BFL3            GHVANPHTAA-AVKR----------KFLRNMSLMFTMEEIENIT----SSLQLDHMKVSH
1SZQ            GHARRRQDGIPKLVD----------KFKINLARQFPTRQQQRILEVSLDRARLEQMPVNE
2HP0            GKIERRIEATPGSLEDPMDDAHLERKFKDCT-AWMPFGESGLLFD----R--LRSLT---
Q6C354.1        GHRQRREEGTPVLLE----------KFARHLRGRFPEGQVEKILAASN--QDIVNMDIDE
                *:  .   .                **       :   :   :         :  :

BFL3            FVDLLVRENIPTLAL
1SZQ            YLDLYVI--------
2HP0            -----ADQGIKTVQP
Q6C354.1        YVDLYVKKD------
                     .
```

Figure S3: Alignment of BFL3 (2-methylcitrate dehydratase) from the byssochlamic acid cluster with several proteins from the MmgE/PrpD family: *E. coli* (1SZQ), *Agrobacterium tumefaciens* (2HPO),[35] and *Yarrowia lipolytica* (Q6C354.1)[11]

7.1.4 Hydrolase 341

The secondary amino acid structure of the hydrolase 341 (BFL1) matches that of the α/β hydrolase superfamily (SSF53474) with the minimal set of α helices and β strands (αA-D and β3-7).[34, 37]. It also has the conserved active site of thiolesterase enzymes, consisting of a catalytic triad: a nucleophile (usually serine), an acidic residue (usually aspartic acid), and a histidine. In BFL1, the nucleophile (S 102) is positioned between the β5 strand and αC helix within the conserved motif GXSXG, the histidine (H 195) after β8 strand and the acidic residue after the β7 strand (D 168), as is standard within the canonical model of thiolesterase enzymes (Figure S4).[37] This evidence suggests that the hydrolase 341 may act as a thiolesterase enzyme, perhaps involved in the release of the polyketide chain from the PKS enzyme.

```
Clustal Omega
BFL1            ---------------------MPQVKLLCLHGAGTSIQILQSQLAPLIRELQKDSTASFHF
Q0C8M2          ----------MRYQASPALVKAPRALLCIHGAGCSPAIFRVQLSKLRAALREN--FEFVY
AF109727        ----MGSDWFRRFGPA---PDSDVRLICLPHAGGAAGAFLALAR----ELT----PEFDV
AAG52991.1      MHRPEAEKWLRRFERA---PDARARLVCLPHAGGSASFFFPLAK----ALA----PAVEV
                                         *:*:  ** :   :          *       .
BFL1            IEGEVECGPGPGIEGIFE--GPYFSFYKWPRTLEDDDESLYDAYGYLSDV----------
Q0C8M2          VTAPFPSSAGPGILPVFADLGPYYSWFESSSDNNHNGPSVSERLAAVHDPIRRTIVDWQT
AF109727        LSVQYPGRQDRRREPPLADIG------------------------LLVDALAGEMAPLA-
AAG52991.1      LAVQYPGRQDRRHEPPVDSIG------------------------GLTNRLLEVLRPFG-
                :            S 102  *                         : :
BFL1            IEEEGPFDGVIGFSHGGTLAFGYLA-----HLAKTRPNDALPFRCAVFMN---APPPFRM
Q0C8M2          QHPHIPIVGAIGFSEGALVTTLLLWQQQMGHLPWLPRMSVALLICPWYQD---EASQYMR
AF109727        DRP----HAFFGHSMGALLAYELAR---------ELRRRALPGPCHLFLSGRFAPTPQGS
AAG52991.1      DRP----LALFGHSMGAIIGYELAL---------RMPEAGLPAPVHLFASGRRAPSRYRD
                 .      . :*.* *. :                            : .
BFL1            D--------TYGN--------------------------------LIVEEGLQDLVRIP
Q0C8M2          NEVMKNHDDDNDS--------------------------------KDTEWQEELVIRIP
AF109727        DSDRLDTDEKVIAMIRRLGGTVGKVFDDPDVMEMVMPPLRADYRAVGAYTWQPGPPLAVP
AAG52991.1      DDVRGASDERLVAELRKLGGSDAAMLADPELLAMVLPAIRSDYRAVETYRHEPGRRVDCP
                :       D 168                       H 195               :  *
BFL1            TLHVVGKKDFIYNFSLKLHELCDSA-NSTLVLHEKGHEIPSDTKN--VNRITKALRELCQ
Q0C8M2          TLHLQGRDDFALAGSKMLVARHFSPREAQVLEFAGQHQFPNRPRD--VLEVINRFRKLCV
AF109727        VTVLVGDQDPVVPVAAAAAWREHTTAGSDLRVLPGGHFYLDQSVPEVAGIVRSALRATAV
AAG52991.1      VTVFTGDHDPRVSVGEARAWEEHTTGPADLRVLPGGHFFLVDQAAPMIATMTEKLA----
                .  . * .*     .        :   : :      *             : . :
BFL1            VSAFL---------------
Q0C8M2          TAQTLE--------------
AF109727        PGPGWRALLHGESPVGTPGPQ
AAG52991.1      -GPALTGSTGGNS--------
                 .
```

Figure S4: Alignment of BFL1 (hydrolase 341) from the byssochlamic acid cluster with the thiolesterases from *Aspergillus terreus* (Q0C8M2), *Streptomyces coelicolor* (AAF43096.2) and *Amycolatopsis mediterranei* (AAG52991.1). Conserved residues from the active site are highlighted in red. The conserved GXSXG motif is highlighted by the red box.

7.1.5 KI-like genes and PEBPs

The function of the remaining proposed core genes, (the ketosteroid isomerase-like genes, *bfL6* and *bfL10*, and the putative PEBP genes *bfL5* and *bfL10*) cannot easily be predicted by bioinformatics, however it is plausible that BFL6 and BFL10 might catalyze an isomerization, and that the putative PEBPs might bind an anion (as is likely to be formed during the dimerisation process).[38] The PEBPs both have a similar predicted secondary structure to that of known PEBPs, but are missing key residues from the conserved DPDXPX$_n$H motif and BFL5 is missing key residues from the GXHR motif (Figure S5).[38, 39]

```
Clustal Omega
BFL5            --------------MIGYIVQFSILLTIHVLAQTPPGYEPSSNHELYLEYPGEISVFPGI
BFL9            MS-ILAYVQYGLGKAFSPIRGH-----DSKAITKTPAFKDIPQPNMTLEAPEC--GPSGS
1KN3_A          -----------MSMWTGPLS-------LHE-------VDEQPQHLLRVTYTEAEVEELGQ
1B7A_A          --------PVDLSKWSGPLS-------LQE-------VDERPQHPLQVKYGGAEVDELGK
Q41261.1        MAAKVSSDPLVIGRVIGDVV-------DHFTSTVKMSVIYNSNNSIKHVYN-------GH
                                . :                       :  :            *
BFL5            SL-------ELSDTKCIP--TLSTLGLSRIQTYIVFLIDID--VILEPNATTILHWYQP-
BFL9            KLLDHHTCLAKDGKGDFPELRWSAPGLGDVKEYVLICEDLDLPIPGLVMHHGIFYGIPPS
1KN3_A          VL-------TPTQVKHRP-GSISWDGLDPGKLYTLILTDPDAPSRKKPVYREWHHFLVV-
1B7A_A          VL-------TPTQVKNRP-TSITWDGLDPGKLYTLVLTDPDAPSRKDPKYREWHHFLVV-
Q41261.1        EL-------FPSAVTSTP--RVEVHGGDMRSFFTLIMTDPDVPGPSDPYLREHLHWIVT-
                 *               *       *    . : :.  * *             :
BFL5            ---------DLKAQDLGSH------LVNTTNNGAAYLGPHPPA-GNTHRYVFLLFQQPQG
BFL9            TTSATNADVQHNGKDAKDYVTTAGWKYIPNMMGSPYLGPAPPLGHGSHRYVFYIVALKEP
1KN3_A          ---------NMKGNDISS-----------GNVLSDYVGSGPPKGTGLHRYVWLVYQQDKP
1B7A_A          ---------NMKGNNISS-----------GTVLSDYVGSGPPKGTGLHRYVWLVYEQEGP
Q41261.1        ---------DIPGTTDSSF----------GKEVVSYE--MPRPNIGIHRFVFLLFKQKKR
                         :  .    .                 *    *      **:*: :
BFL5            YTLPSCFSTIFPETVEARAGFNIDEFIEVAGLGDVVAANYVNVTNPATPSTTLTATTTSL
BFL9            LDPEQ--PEKLNRQT-----LA------EAMTGKVIGWGQWIGTWERPWPR---------
1KN3_A          LRCD--EPILTNRSGDHRGKFKTAAFRKKYHLGAPVAGTCYQAEWDSYVPKLYKQL----
1B7A_A          LKCD--EPILSNRSGDHRGKFKVASFRKKYELGAPVAGTCYQAEWDDYVPKLYEQL----
Q41261.1        GQAM------LSPPVVCRDGFNTRKFTQENELGLPVAAVFFNCQRETAARRR--------
                                    :           *  :.
BFL5            STAPCPITMAKFLSST
BFL9            ----------------
1KN3_A          SGK-------------
1B7A_A          SGK-------------
Q41261.1        ----------------
```

Figure S5: Alignment of BFL5 and BFL9 with PEBPs from *Mus musculus* (1KN3_A), *Bos taurus* (1B7A_A) and *Antirrhinum majus* (Q41261.1). Key conserved residues are highlighted in red. The DPDXPXnH and GXHR motifs are highlighted by red boxes.

7.1.6 Promoter region of *bfR4*

The BGCs from *B. fulva* IMI 40021 and *B. fulva* IMI 58422 responsible for byssochlamic acid biosynthesis are over 97% identical, however there is a significant difference (an inversion) in the promoter region of *bfR4*, a putative pathway specific activator (Figure S6). This variance may explain the differences in byssochlamic acid production between the two strains.

```
Clustal Omega

IMI40021      TTTGGTAATCAGACAAATGCTTTGTGCAGCAAAGATGGTTTTCCTATAACCCGTCATGTG
IMI58422      TTTGGTAATCAGACAAATGCTTTGTGCAGCAAAGATGGTTTTCCTATAACCCGTCATGTG
              ************************************************************

IMI40021      TCAATTAATTACATCTTATCACTTGCTATATCATTTTCAATTGCCCTGGTCCAAGAGGCA
IMI58422      TCAATCAATTACATCTTATCACTTGCTATATCATTTTCAATTGCCTCTTGGACCAGGGCA
              ***** ***************************************           ****

IMI40021      ATTGAAAATGATATATTAAGTGTGGAAAATATTTGGTGCTGCCCCCATCGGCAGCGGAGC
IMI58422      ATTGAAAATGATATATTAAGTGTGGAAAACATTTGGTGCTGCCCCCATCGGCAGCGGAGC
              ***************************** ******************************

IMI40021      TAAAGGGCCCCTTAACTCCGAAATCATGACATCCCGTGCGACAACTCTGAAAGTCAACTT
IMI58422      TAAAGGGCCCCTTAACTCCGAAATCATGACATCCCGTGCGACAACTCTGAAAGTCAACTT
              ************************************************************

IMI40021      ACCCTCGCGGTAGAGTATGCAATCCGCTGAATCGAGCGTGCGAAAGCATGCGTCTGATCC
IMI58422      ACCCTCGCGGTAGAGTATGCAATCCGCTGAATCGAGCGTGCGAAAGCATGCGTCTGATCC
              ************************************************************

IMI40021      TTATTTGCTCATCACTATCGTTGCGTTCAAAGGCACATGAGAAGCATGGAGTCTGCTGTC
IMI58422      TTATTTGCTCATCACTATCGTTGCGTTCAAAGGCACATGAGAAGCATGGAGTCTGCTGTC
              ************************************************************
```

Figure S6: Alignment of *bfR4* from *B. fulva* strains IMI 40021 and IMI 58422. The beginning of the first exon from the putative pathway specific activator is highlighted in blue. The significant difference between the two promoter regions is highlighted by the red box.

8. Comparison of maleidride-type clusters

8.1 Artemis comparison

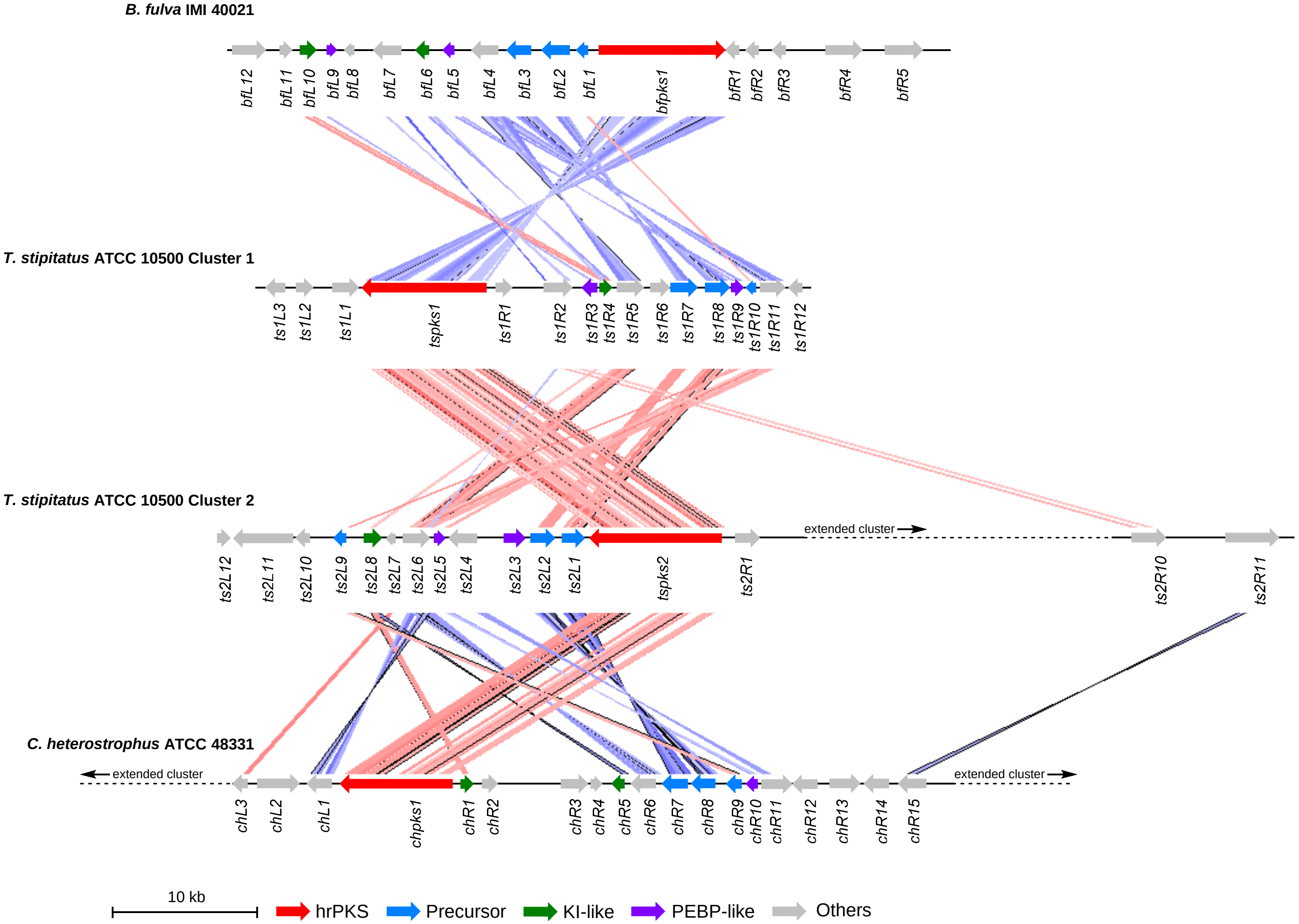

Figure S7: Comparison of the byssochlamic acid 4 cluster (top), to the putative maleidride clusters from *Talaromyces* and *Cochliobolus* using ACT.[40] Regions of homology are identified by pink and blue lines. Pink lines denote homology on the same strand, and blue strands denote homology on the opposite strand.

8.2 Homology comparison tables

Percentage identity matrices were generated to compare the core genes common to all maleidride-type clusters.[41]

Table S3: Percentage identity matrix of PKS protein sequences from the byssochlamic acid cluster, the two *T. stipitatus* clusters and the *C. heterostrophus* cluster

	BFpks1	TSpks1	TSpks2	CHpks1
**BFpks1**	**100**	45.82	47	50.71
**TSpks1**	45.82	**100**	56.51	45.4
**TSpks2**	47	56.51	**100**	45.2
**CHpks1**	50.71	45.4	45.2	**100**

Table S4: Percentage identity matrix of hydrolase 341 protein sequences from the byssochlamic acid cluster, the two *T. stipitatus* clusters and the *C. heterostrophus* cluster

	BFL1	TS1R10	TS2L9	CHR9
**BFL1**	**100**	44.8	53.85	54.34
**TS1R10**	44.8	**100**	46.15	41.55
**TS2L9**	53.85	46.15	**100**	51.6
**CHR9**	54.34	41.55	51.6	**100**

Table S5: Percentage identity matrix of citrate synthase-like protein sequences from the byssochlamic acid cluster, the two *T. stipitatus* clusters and the *C. heterostrophus* cluster

	BFL2	TS1R7	TS2L2	CHR8
**BFL2**	**100**	50.24	55.33	63.95
**TS1R7**	50.24	**100**	45.31	43.76
**TS2L2**	55.33	45.31	**100**	58.28
**CHR8**	63.95	43.76	58.28	**100**

Table S6: Percentage identity matrix of 2-methyl citrate dehydratase protein sequences from the byssochlamic acid cluster, the two *T. stipitatus* clusters and the *C. heterostrophus* cluster

	BFL3	TS1R8	TS2L1	CHR7
**BFL3**	**100**	52.16	63.52	61.07
**TS1R8**	52.16	**100**	54	50.1
**TS2L1**	63.52	54	**100**	58.81
**CHR7**	61.07	50.1	58.81	**100**

Table S7: Percentage identity matrix of ketosteroid isomerase-like protein sequences from the byssochlamic acid cluster, the two *T. stipitatus* clusters and the *C. heterostrophus* cluster

	BFL6	BFL10	TS1R4	TS2L8	CHR1
**BFL6**	**100**	49.78	38.81	39.48	51.28
**BFL10**	49.78	**100**	40.49	43.98	46.08
**TS1R4**	38.81	40.49	**100**	35.55	34.72
**TS2L8**	39.48	43.98	35.55	**100**	39.47
**CHR1**	51.28	46.08	34.72	39.47	**100**

Table S8: Percentage identity matrix of PEBP sequences from the byssochlamic acid cluster, the two *T. stipitatus* clusters and the *C. heterostrophus* cluster

	BFL5	BFL9	TS1R3	TS1R9	TS2L3	TS2L5	CHR10
**BFL5**	**100**	20.38	17.42	36.41	21.35	41.63	43.48
**BFL9**	20.38	**100**	38.05	20.37	20.95	18.79	21.05
**TS1R3**	17.42	38.05	**100**	16.98	15.75	14.63	19.33
**TS1R9**	36.41	20.37	16.98	**100**	20.59	37.63	34.64
**TS2L3**	21.35	20.95	15.75	20.59	**100**	22.29	20.79
**TS2L5**	41.63	18.79	14.63	37.63	22.29	**100**	38.61
**CHR10**	43.48	21.05	19.33	34.64	20.79	38.61	**100**

9. Primer table

Table S9: Primers (all primers were synthesized by Sigma)

Primer Name	Sequence 5' to 3'
BFIMI40021-PKS-P1-F	ATGCCAACTTTGTACAAAAAAGCAGGCTCCATGACGTGCAAGGATAAGAA
BFIMI40021-PKS-P2-R	TAGTGCTCCAATCGTGTAGG
BFIMI40021-PKS-P3-F	GTGCATACCTTGAGAGTCTGG
BFIMI40021-PKS-P4-R	GAGTAGCCACCTTGAGCTTC
BFIMI40021-PKS-P5-F	CAGAAGATCCTATAAGGCAGG
BFIMI40021-PKS-P6-R	AATGCCAACTTTGTACAAGAAAGCTGGGTCTCACTCCTTTTCAGCATCTA
BFIMI40021-Hyd341-F	TTTCTTTCAACACAAGATCCCAAAGTCAAAATGCCACAGGTGAAGTTACT
BFIMI40021-Hyd341-R	TTCATTCTATGCGTTATGAACATGTTCCCTTTAGAGAAATGCTGATACTT
BFIMI40021-CS-F	AACAGCTACCCCGCTTGAGCAGACATCACCATGTCTTCTGGTACACTTTT
BFIMI40021-CS-R	ACGACAATGTCCATATCATCAATCATGACCTTATAGCTTAGGCGAGAAGT
BFIMI40021-MCD-F	TCGACTGACCAATTCCGCAGCTCGTCAAAGATGTCTACAGAGAAGTACGA
BFIMI40021-MCD-R	GGTTGGCTGGTAGACGTCATATAATCATACCTAGAGCGCTAAAGTTGGTA
BFIMI40021-KI1-F	AACAGCTACCCCGCTTGAGCAGACATCACCATGGTGTCGTCCAAGGTTTT
BFIMI40021-KI1-R	AGGTTGGCTGGTAGACGTCATATAATCATACTAATAATGACCATATTTGG
BFIMI40021-KI2-F	TTTCTTTCAACACAAGATCCCAAAGTCAAAATGGTGGCAATGTTTATTAC
BFIMI40021-KI2-R	TTCATTCTATGCGTTATGAACATGTTCCCTTCAATCTATATCTGGTGGCA
BFIMI40021-PEBP1-F	AACAGCTACCCCGCTTGAGCAGACATCACCATGATAGGATACATTGTCCA
BFIMI40021-PEBP1-R	GGTTGGCTGGTAGACGTCATATAATCATACTCATGTCGAAGACAGAAATT
BFIMI40021-PEBP2-F	TTTCTTTCAACACAAGATCCCAAAGTCAAAATGAGCATCCTCGCTTATGT
BFIMI40021-PEBP2-R	TTCATTCTATGCGTTATGAACATGTTCCCTTTACCTTGGCCATGGACGCT
pTY-F1	GATTGTACTGAGAGTGCACC
Padh-mid/Tadh-mid-R	CACGAGGTAAACGAAACGCTATTAAATTAAAGAGAGGGAAAGGAAAGGTC
Teno-mid/PgpdA-mid-F	TACCGAGCTCCCAAATCTGTCCAGATCATGTGACGATTCGATTAGGCCAG
pTY-R1	ACTCTAGAGTCGACCTGCAG
BFIMI40021-PKSKO-P1-F	TATCGGAAGCTCGAGAAAGC
BFIMI40021-PKSKO-P2-R	CACTAGAGGATCCCCATCATGTGCCAAGTCGTCTAGGAATC
BFIMI40021-PKSKO-P3-F	CACATCTCCACTCGACCTGCCTGTCTGTGAGTTCAAGAG
BFIMI40021-PKSKO-P4-R	CCAGTTACGAAGTTCGACAGC
HygRP5-F	CATGATGGGGATCCTCTAGTG
HygRP6-R	CGTCAGGACATTGTTGGAG
HygRP7-F	GCTTTCAGCTTCGATGTAGG
HygRP8-R	CAGGTCGAGTGGAGATGTG
BF-KI1-F	GGTTTTAATACGGGCACTT
BF-KI1-R	CAAGGTGTTTATAGGAGTCGT
BF-KI2-F	ACTTTGAGAATCCAAGCCT
BF-KI2-R	TAACCTACGGACGTCGTTC
byssPKS-test1-PF	CCGATGCTATGGGAGATAAG
pgpdA-Bg-R	GCTCGACGTATTTCAGTGTC
TtrpC-Nd-F	GCTCCGTAACACCCAATACG
byssPKS-test2-PR	AGAAGCTCCAGAATGGGTAC
PgpdA-Nd-R	GGATGGAGTATGGATGTAGC

10. Plasmid construction

10.1 pE-YA-bfpks1

The *bfpks1* gene was amplified from cDNA as three overlapping fragments using primer sets BFIMI40021-PKS-P1-F and –P2-R, BFIMI40021-PKS-P3-F and –P4-R, and BFIMI40021-PKS-P5-F and –P6–R. BFIMI40021-PKS-P1-F and BFIMI40021-PKS-P6-R contained 30 base tails homologous to the cut ends of the vector pE-YA digested with *Not*I and *Asc*I (Figure S8). The gene was reassembled in pE-YA via homologous recombination in the yeast *Saccharomyces cerevisiae*.[42] pE-YA is a Gateway modified vector, which allows the assembled gene to be transferred to any Gateway destination vector via LR recombination (Figure S8).[43]

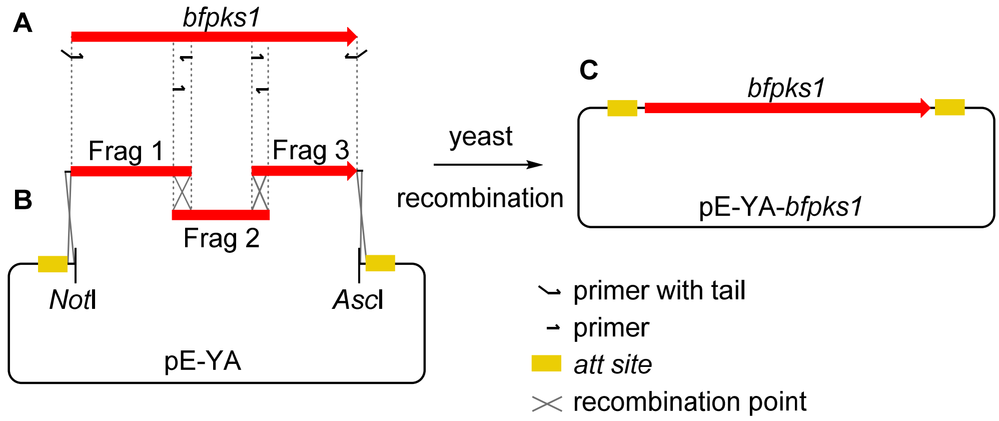

Figure S8: Assembly by yeast recombination and PCR of the *bfpks1* gene into the Gateway Entry vector, pE-YA. ***A,*** cDNA of the *bfpks1* gene with position of the primers used to amplify the fragments 1, 2 and 3. ***B,*** Fragment 1 and fragment 3 contain 30 bp tails homologous to the cut ends of pEYA, digested with *Not*I and *Asc*I. ***C,*** Yeast recombination reassembled the complete *bfpks1* gene between the *att* recombination sites allowing transfer of the gene to a destination vector via LR recombination.

10.2 pTYarg-BF-PMCH

The hydrolase 341 (*bfL1*), the citrate synthase-like gene (*bf*L2), and the 2-methylcitrate dehydratase gene (*bfL3*) were amplified from cDNA and recombined directly into the arginine selection multi-gene expression plasmid, pTYarg-GS under the control of P_{adh},[44] P_{gpdA}[45] and P_{eno}[44] promoters respectively. *bfL1* was amplified using primers BFIMI40021-Hyd341-F and –R. *bf*L2 was amplified using primers BFIMI40021-CS-F and –R. *bfL3* was amplified using primers BFIMI40021-MCD-F and -R. pTYarg-GS was digested with *Asc*I prior to recombination. After *bfL1, bf*L2 and *bfL3* were cloned into the plasmid, the Gateway cassette was then replaced with the *bfpks1* gene via LR recombination, placing the PKS gene under the control of the inducible *amyB* promoter (P_{amyB}),[46] to create plasmid pTYarg-BF-PMCH (Figure S9).

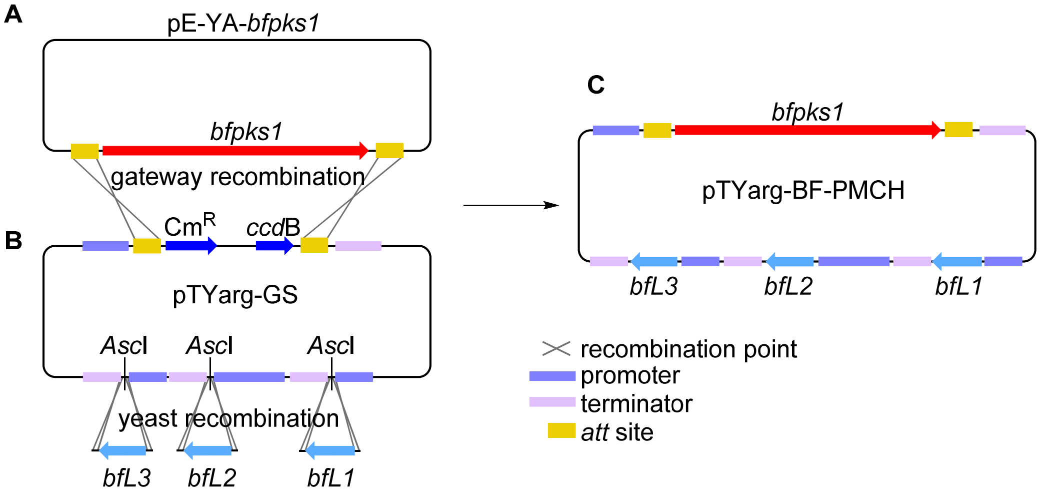

Figure S9: Construction of pTYarg-BF-PMCH via yeast recombination and LR recombination. ***A,*** pE-YA-*bfpks1* plasmid constructed previously. ***B,*** The *bfpks1* gene was transferred site specifically and directionally via LR recombination into the multi-gene expression vector pTYarg-GS. The three further monomer genes (*bfL1, bf*L2 and *bfL3*) were cloned into the multi-gene expression vector via yeast recombination. Each gene was amplified with primers containing 30 base tails homologous to the specific *Asc*I cut ends of the plasmid. ***C*** The complete plasmid contains all four monomer producing genes under the control of strong *Aspergillus* promoters.

10.3 pTYade-GS-KIs

The two KI-like genes (*bfL6* and *bfL10*), were amplified from cDNA and cloned into pTYade-GS[47] under the control of the P_{adh} and P_{gpdA} promoters respectively. *bfL6* was amplified using primers BFIMI40021-KI1-F and –R. *bfL10* was amplified using primers BFIMI40021-KI2-F and –R. Both genes were recombined into *Asc*I digested pTYade-GS to produce plasmid pTYade-GS-KIs (Figure S10).

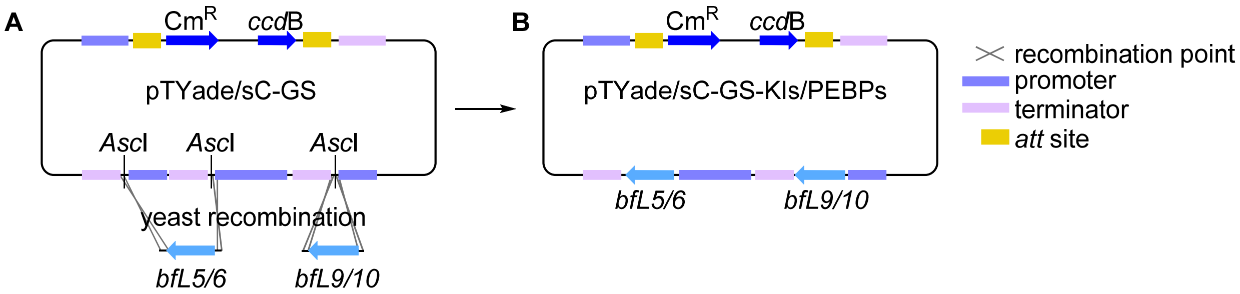

Figure S10: Construction of pTYade-GS-KIs and pTYsC-GS-PEBPs via yeast recombination. ***A,*** Either the KIs or the PEBPs were recombined into either *Asc*I digested pTYade-GS or *Asc*I digested pTYsC-GS. ***B,*** The complete plasmids contain either both KIs or both PEBPs under the control of strong *Aspergillus* promoters.

10.4 pTYsC-GS-PEBPs

The two PEBP genes (*bfL5* and *bfL9*), were amplified from cDNA and cloned into pTYsC-GS[47] under the control of the P_{adh} and P_{gpdA} promoters respectively. *bfL5* was amplified using primers BFIMI40021-PEBP1-F and –R. *bfL10* was amplified using primers BFIMI40021-PEBP2-F and –R. Both genes were recombined into *Asc*I digested pTYsC-GS to produce plasmid pTYsC-GS-PEBPs (Figure S10).

10.5 pTYade-GS-KI1 and pTYade-GS-KI2

The plasmid pTYade-GS-KIs was cut with *Bsp*TI (for the KI1 deletion) or *Psh*AI (for the KI2 deletion). Deletion fragments were amplified from the plasmid pTYade-GS using primers Teno-mid/PgpdA-mid-F and pTY-R1 (for KI1 deletion) and primers pTY-F1and Padh-mid/Tadh-mid-R (for KI2 deletion). Yeast recombination produced the plasmids pTYade-GS-KI1 and pTYade-GS-KI2 (Figure S11).

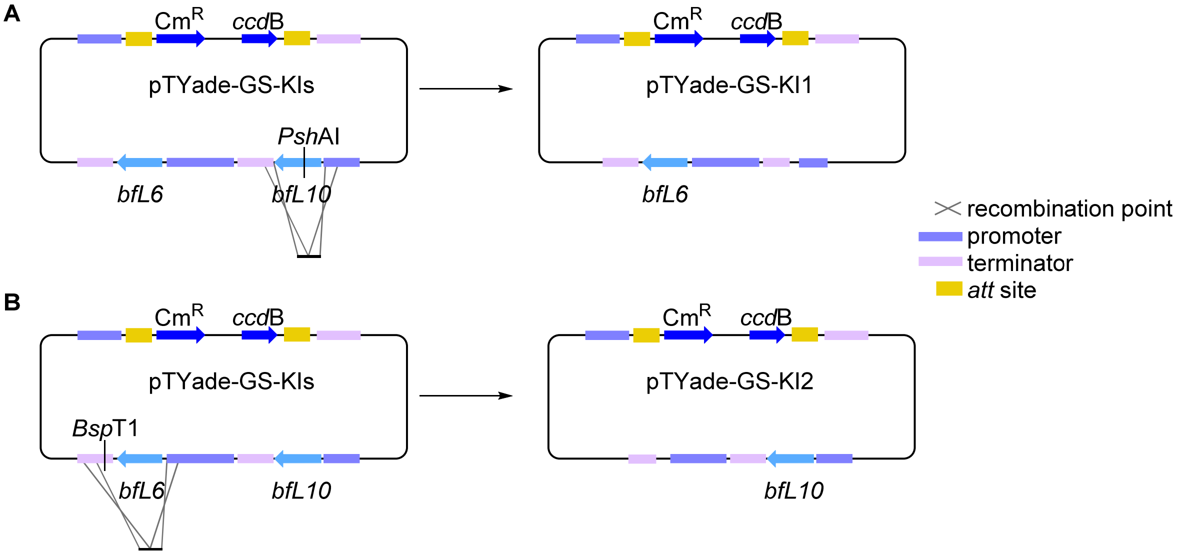

Figure S11: Construction of the plasmids pTYade-GS-KI1 and pTYade-GS-KI2 by yeast recombination. ***A,*** pTYade-GS-KIs was cut with *Psh*AI (within the KI2 gene). A deletion fragment was amplified from pTYade-GS. Yeast recombination produced plasmid pTYade-GS-KI1. ***B,*** pTYade-KIs was cut with *Bsp*TI (within the terminator region of the KI1 cassette). A deletion fragment was amplified from pTYade-GS. Yeast recombination produced plasmid pTYade-GS-KI2.

11. Transformation

11.1 *S. cerevisiae*

Transformation was performed using the LiAc PEG mediated method.[48] Approximately 0.5 - 1.0 µg of each DNA fragment was added, with linear DNA fragments to be joined containing at least 30 bp overlap.

11.2 *A. oryzae*

Transformation was performed using the PEG mediated protoplast method. *A. oryzae* NSAR1 was grown on MEA plates for 1-2 weeks. Spores from a single plate were inoculated into 100 ml of GN medium (2 % *w/v* D(+)-glucose monohydrate 1 % *w/v* Nutrient broth Nr. 2) and cultured overnight at 28 °C with shaking at 200 rpm. Germinated spores were pelleted and washed with sterile H_2O and then 0.8 M NaCl. The pellet was then resuspended in 10 ml filter sterilized protoplasting solution (0.8M NaCl, 5mg/ml driselase (Sigma), 20mg/ml *Trichoderma* lysing enzyme (Sigma)), and mixed gently at 25 °C for 90-120 min. Protoplasts were separated from the mycelium through sterile miracloth and centrifuged at 1000 x g for 3 min. The pellet was washed with solution 1 (0.8M NaCl, 50 mM $CaCl_2$ 10 mM Tris-HCl [pH 7.5]), and then resuspended in 200-500 µl of solution 1, and for each transformation, 100 µl was transferred to a 50 ml centrifuge tube on ice. 5-10 µg (10 µl max) of plasmid DNA was added to the protoplasts and gently mixed. The tube was incubated on ice for 2 min, after which 1 ml of solution 2 (60% *w/v* PEG 3350, 0.8 M NaCl, 10 mM $CaCl_2$, 50 mM Tris-HCl pH 7.5) was added and the tube was incubated at room temperature for 20 min. 40 ml of molten (50 °C) CZD/S top medium with appropriate supplements (3.5% *w/v* Czapek Dox broth, 1 M sorbitol, 0.8% *w/v* agar) was added and gently mixed. 10 ml each of the mix was overlaid onto four plates prepared with appropriate supplements (15 ml of 3.5% *w/v* Czapek Dox broth, 1 M sorbitol, 1.5% *w/v* agar). The appropriate supplements depended on the plasmid(s) undergoing transformation: 0.005% *w/v* adenine, 0.01% *w/v* methionine, 0.2% *w/v* ammonium sulfate. Plates were then incubated at 28 °C for 3-5 days until colonies appeared. Colonies were transferred to secondary plates (3.5% *w/v* Czapek Dox broth, 1.5% *w/v* agar and the appropriate supplements). After sporulation had occurred, the spores were streaked to single colonies on tertiary plates, and finally single colonies were transferred to quaternary plates to ensure genetic purity.

11.3 *B. fulva* IMI40021

Transformation was performed using the PEG mediated protoplast method. *B. fulva* IMI 40021 was grown on PDA plates for 1-2 weeks. Spores from a single plate were inoculated into 100 ml PDB and cultured overnight at 25 °C with shaking at 200 rpm. Germinated spores were pelleted and washed with sterile H_2O and then 0.7 M KCl. The pellet was then resuspended in 10 ml filter sterilized protoplasting solution (0.7 M KCl, 5 mg/ml driselase (Sigma), 5 mg/ml *Trichoderma* lysing enzyme (Sigma)), and mixed gently at 25 °C for 90-120 min. Protoplasts were separated from the mycelium through miracloth and centrifuged at 1000 x g for 3 min. The pellet was washed with solution 1 (0.7 M KCl, 50 mM $CaCl_2$ 10 mM Tris-HCl [pH 7.5]), and then resuspended in 400 µl of solution 1. Aliquots of 200 µl were taken for each transformation and added to 50 µl of PEG solution (25% *w/v* PEG 3350, 0.7 M KCl, 50 mM $CaCl_2$ 10 mM Tris-HCl [pH 7.5]) along with ~50 ng DNA of each knock-out fragment. The protoplasts were gently mixed and placed on ice for 20 min. Subsequently, 500 µl of PEG solution was added and incubated at room temperature for 5 min. Aliquots of 175 µl were spread onto plates containing PDA + 1M sorbitol, and the plates incubated at 25 °C overnight. Overlays of ~10 ml PDA containing hygromycin to produce a final concentration of 100 µg/ml were added the next day. Transformants appeared approximately 3-5 days later, and were subcultured onto secondary plates containing 100 µg/ml hygromycin. After sporulation had occurred, the spores were streaked to single colonies on tertiary plates, and finally single colonies were transferred to quaternary plates to ensure genetic purity.

12. *bfpks1* gene disruption

The byssochlamic acid PKS was knocked-out using the bipartite method.[49] This method relies on the splitting of the resistance marker (with a ~500 bp overlap), which leads to the requirement for homologous recombination between the two resistance marker halves for selection to occur. This activation of the homologous recombination pathway appears to lead to higher gene disruption levels.

The hygromycin resistance cassette was utilized in the transformation of *B. fulva* IMI 40021 which consists of the *gpdA* promoter, the *HygR* resistance gene and the *trpC* terminator.[50] The gene knock-out fragments, which consisted of a region homologous (~1 - 2 kb) to the *B. fulva* IMI 40021 genome and one half of the resistance cassette, were amplified by several rounds of PCR. The left hand side homologous region was amplified from *B. fulva* IMI 40021 gDNA using primers BFIMI40021-PKSKO-P1-F and –P2-R. The reverse primer contained a tail homologous to the beginning of the gpdA promoter. The right hand side homologous region was amplified from the same template using primers BFIMI40021-PKSKO-P3-F and –P4-R. The forward primer contained a tail homologous to the *trpC* terminator. The split fragments of the resistance cassette were amplified separately using primers HygRP5-F and –P6-R, and HygRP7-F and –P8-R. Subsequently approximately equal amounts of the left hand side homologous region and the left hand side of the resistance cassette (and vice versa) were mixed and used as a template in a fusion PCR amplification using primers BFIMI40021-PKSKO-P1-F and HygRP6-R, and HygRP7-F and BFIMI40021-PKSKO-P4-R. Approximately equal amounts of the fused PCR products were used in a transformation of *B. fulva* IMI 40021.

12.1 Genetic characterization of gene disruption

Transformants from the gene knock-out experiment were inoculated from a single plate into 100 ml CGB and cultured at 25 °C. After 2-3 days, 10 ml of the culture was removed, centrifuged and lyophilized for gDNA extraction. Integration of the knock-out construct was tested via primers designed outside the homologous regions, and within the resistance cassette. The primers used were byssPKS-test1-PFand PgpdA-Bg-R (1981 bp) and TrpC-Nd-F and byssPKS-test2-PR (1854 bp) (Figure S12). Transformants that had been determined to be knock-outs were then subjected to metabolite extraction using the remaining 90 ml of culture after 12 days incubation.

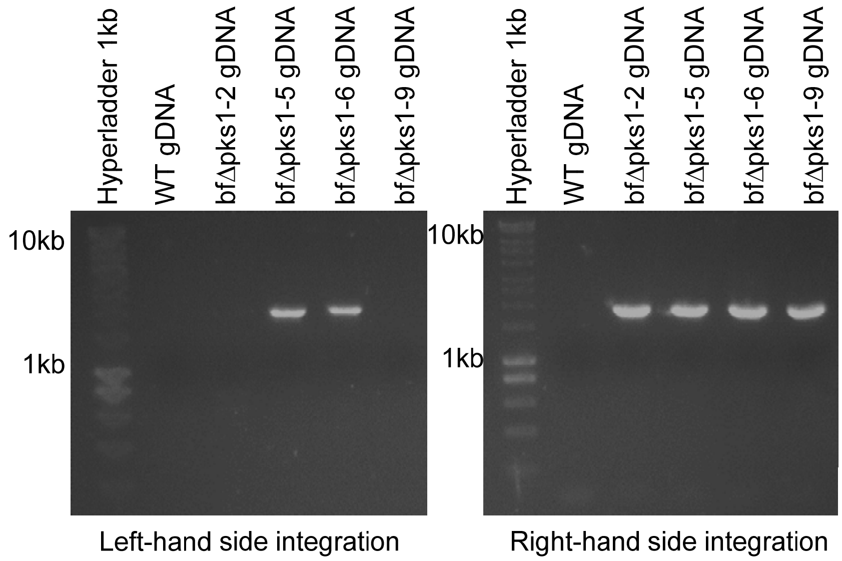

Figure S12: PCR analysis of *bfΔpks1* strains

13. Metabolite extraction and LCMS analysis

13.1 *B. fulva* IMI 40021

Metabolite extraction was performed after 12 days. The culture liquid was separated from the mycelium, and an equal volume of ethyl acetate was added. The mixture was acidified with HCl to pH 4.0 and mixed. The ethyl acetate phase was separated and dried ($MgSO_4$), then resuspended in acetonitrile at 5 mg/ml.

13.2 LCMS analysis of standards

A homologue of the monomer **10** was previously synthesized. It undergoes spontaneous decarboxylation to **9**.[51] The UV absorbances of **9** and **10**, and the mass fragmentation pattern of **10** are shown in Figure S13:

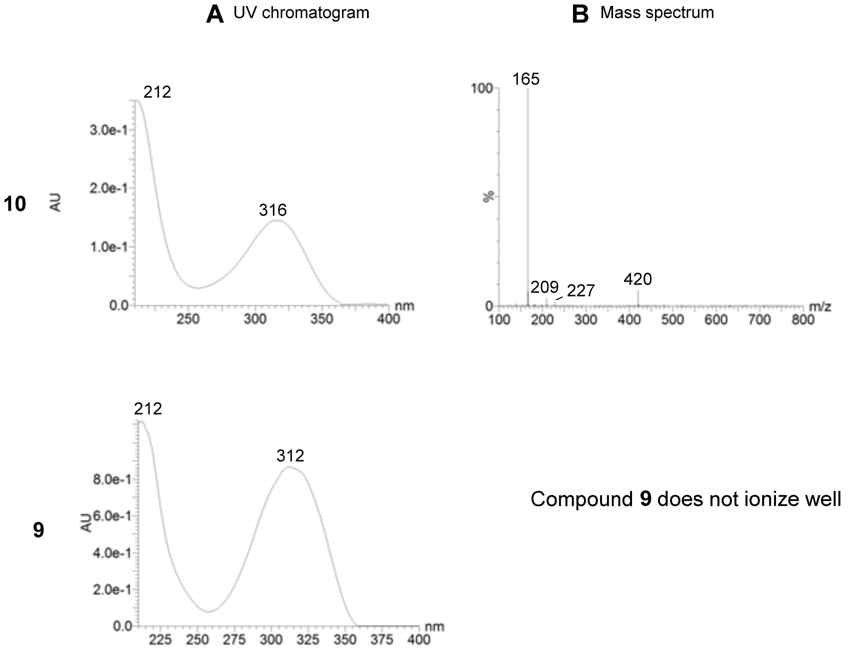

Figure S13: LCMS characteristics of synthetic homologues of **9** and **10.** ***A,*** UV absorbances for compounds **9** and **10**. ***B,*** ES- mass fragmentation pattern for compound **10**. Compound **9** does not ionize well.

Byssochlamic acid **1** and agnestadride A **8** have been previously purified and fully characterized.[51] Their LCMS characteristics are shown in Figure S14

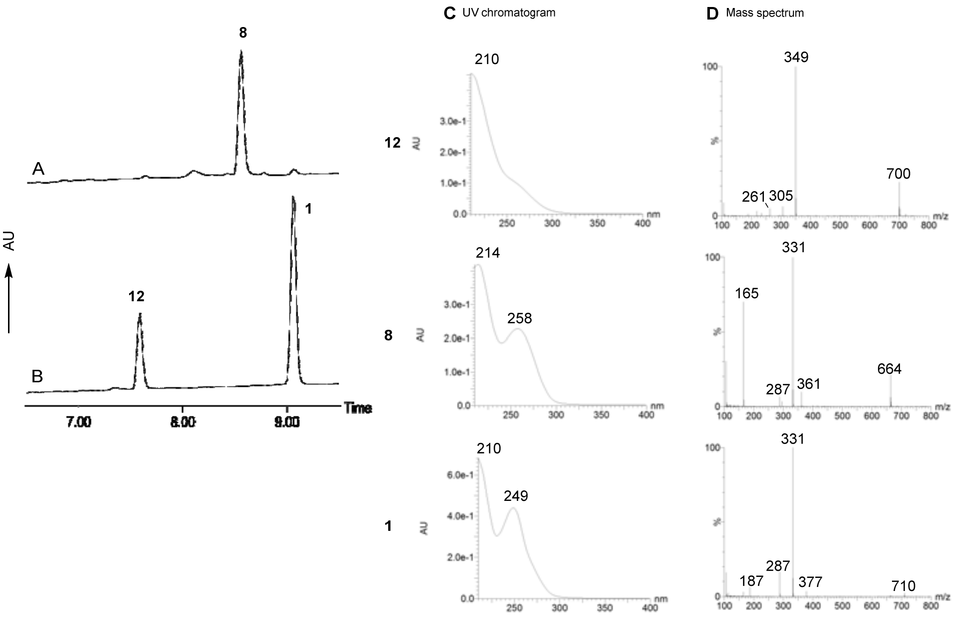

Figure S14: LCMS analysis of standards byssochlamic acid **1** and agnestadride A **8**. ***A,*** HPLC (DAD) of purified byssochlamic acid **1** and compound **12**. ***B,*** HPLC (DAD) of purified agnestadride A **8**. ***C,*** UV absorbances for compounds **1**, **8** and **12**. ***D,*** ES- mass fragmentation pattern for compounds **1**, **8** and **12**.

13.3 LCMS analysis of *B. fulva* strains

The peaks identified from the *B. fulva* IMI 40021 WT strain were characterized by their retention time, UV absorbances and mass fragmentation patterns. None of the maleidride related compounds were identified in the *bfΔpks1* strains (Figure S15).

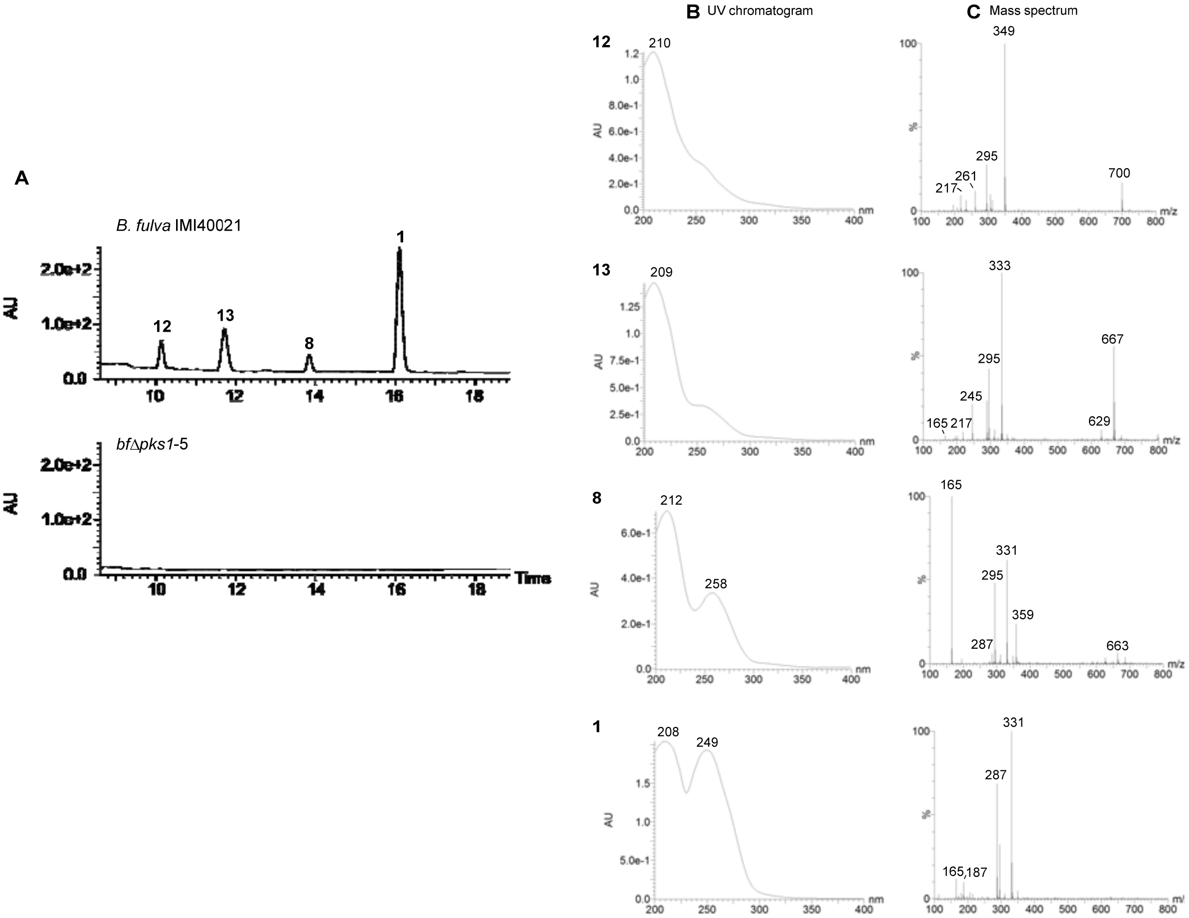

Figure S15: ***A,*** HPLC (DAD) analysis of comparison between *B. fulva* IMI 40021 WT and the *bfΔpks1*-5 strain. ***B,*** UV absorbances for the compounds identified from the *B. fulva* IMI 40021 strain ***C,*** ES- mass fragmentation patterns for the compounds identified from the *B. fulva* IMI 40021 strain.

13.4 Metabolite extraction and LCMS analysis of *A. oryzae* NSAR1

Metabolite extraction was performed after 7 days. The culture liquid and mycelium were blended with an equal volume of ethyl acetate. The mixture was acidified with HCl to pH 4.0 and mixed. The mycelium was removed by vacuum filtration. The ethyl acetate phase was separated and dried ($MgSO_4$), then resuspended in acetonitrile at 5 mg/ml.

13.5 LCMS analysis of *A. oryzae* NSAR1 transformants

13.5.1 Comparison between *A. oryzae* strains

A search for the 331 ion in the ES- spectrum revealed the presence of peaks with the same retention time as the previously purified and fully characterized byssochlamic acid **1** and agnestadride A **8** in strains AO-BF-PMCH+KIs and AO-BF-PMCH+KIs+PEBPs (Figure S16).[51] Strain AO-BF-PMCH+KIs expressed the *B. fulva* genes *bfpks1, bfL1, bfL2, bfL3, bfL6* and *bfL10*. Strain AO-BF-PMCH+KIs+PEBPs expressed the *B. fulva* genes *bfpks1, bfL1, bfL2, bfL3, bfL6, bfL10, bfL5* and *bfL9*.

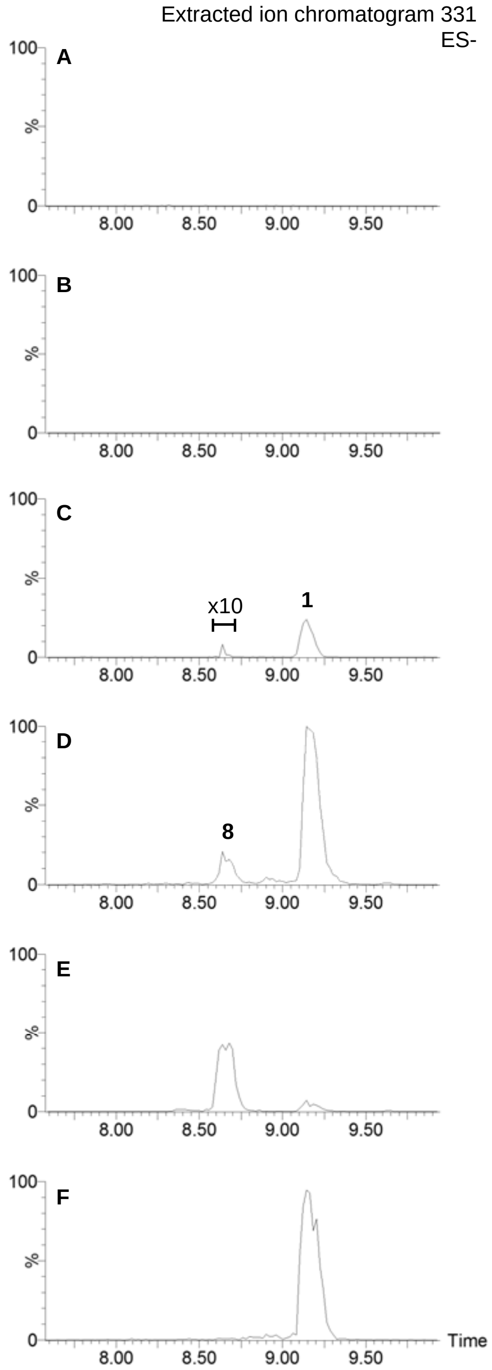

Figure S16: Extracted ion chromatogram searching for 331 in the ES- (the masses of byssochlamic acid and agnestadride A are both 332). ***A,*** extract from untransformed *A. oryzae* NSAR1. ***B,*** extract from *A. oryzae* transformant BF-PMCH. ***C,*** extract from *A. oryzae* transformant BF-PMCH+KIs. Compound **8** eluting at 8.6 min is magnified 10x. ***D,*** extract from *A. oryzae* transformant BF-PMCH+KIs+PEBPs. ***E,*** purified agnestadride A **8**. ***E,*** purified byssochlamic acid **1**.

13.5.2 LCMS analysis of strain AO-BF-PMCH

Strain AO-BF-PMCH expressed the *B. fulva* genes *bfpks1, bfL1, bfL2* and *bfL3.* Two novel peaks were identified in the extracts of AO-BF-PMCH compared to crude extract from untransformed *A. oryzae* NSAR1, which correspond to compounds **9** and **10**. The UV absorbances of compounds **9** and **10**, and the mass fragmentation pattern of compound **10** are shown in Figure S17.

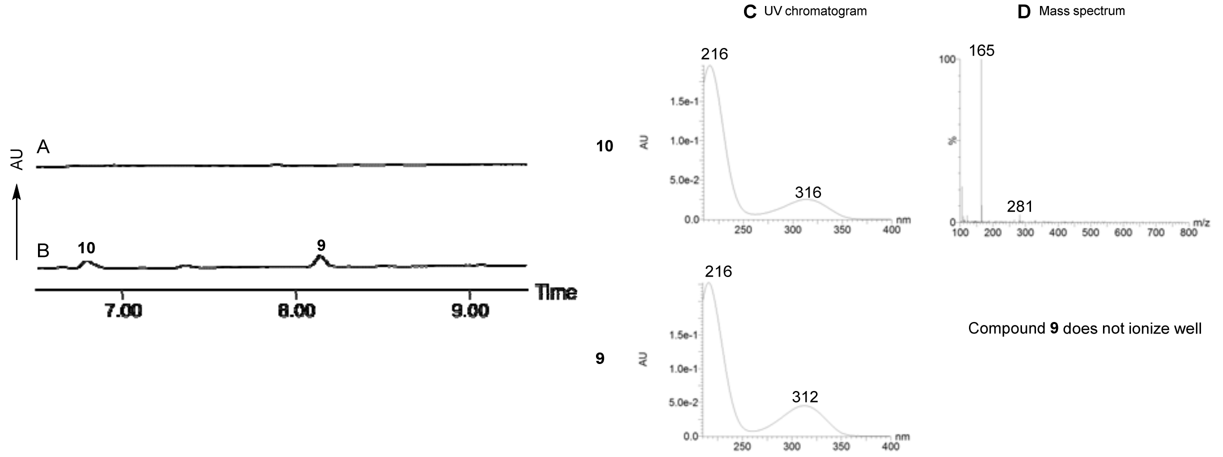

Figure S17: LCMS analysis of strain AO-BF-PMCH. ***A,*** HPLC (DAD) of crude extract from untransformed *A. oryzae* NSAR1. ***B,*** HPLC (DAD) of crude extract from AO-BF-PMCH with two novel peaks identified. ***C,*** UV absorbances for compounds **9** and **10**. ***D,*** ES- mass fragmentation pattern for compound **10**. Compound **9** does not ionize well.

13.5.3 LCMS analysis of strain AO-BF-PMCH+KIs

Strain AO-BF-PMCH+KIs expressed the *B. fulva* genes *bfpks1, bfL1, bfL2, bfL3, bfL6* and *bfL10.* Novel peaks were identified in this strain compared to crude extract from untransformed *A. oryzae* NSAR1. Their UV absorbances and mass fragmentation patterns are shown in Figure S18.

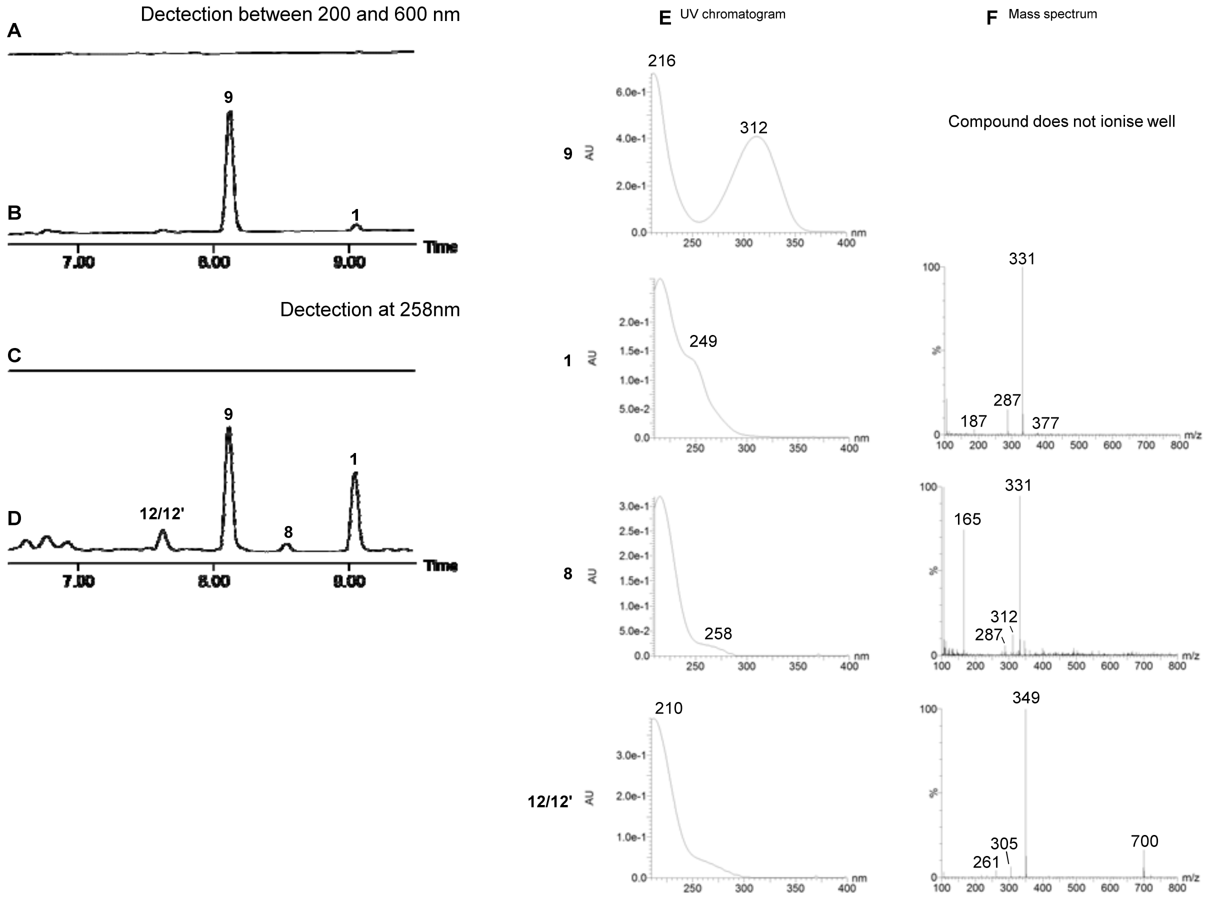

Figure S18: LCMS analysis of strain AO-BF-PMCH+KIs. ***A,*** HPLC (DAD) of crude extract from untransformed *A. oryzae* NSAR1. ***B,*** HPLC (DAD) of crude extract from AO-BF-PMCH+KIs with two novel peaks identified. ***C,*** HPLC (DAD at 258nm) of crude extract from untransformed *A. oryzae* NSAR1. ***D,*** HPLC (DAD at 258nm) of crude extract from AO-BF-PMCH+KIs with four novel peaks identified. ***E,*** UV absorbances for compounds **9**, **1**, **8** and **12/12'**. ***F,*** ES- mass fragmentation patterns for compounds **1**, **8** and **12/12'**. Compound **9** does not ionize well.

13.5.4 LCMS analysis of AO-BF-PMCH+KI1/2 strains

Strain AO-BF-PMCH+KI1 expressed the *B. fulva* genes *bfpks1, bfL1, bfL2, bfL3* and *bfL6.* Strain AO-BF-PMCH+KI2 expressed the *B. fulva* genes *bfpks1, bfL1, bfL2, bfL3* and *bfL10.* Crude extracts from these strains only showed production of varying amounts of **9** and **10**, there was no evidence for any dimerized products (Figure S19).

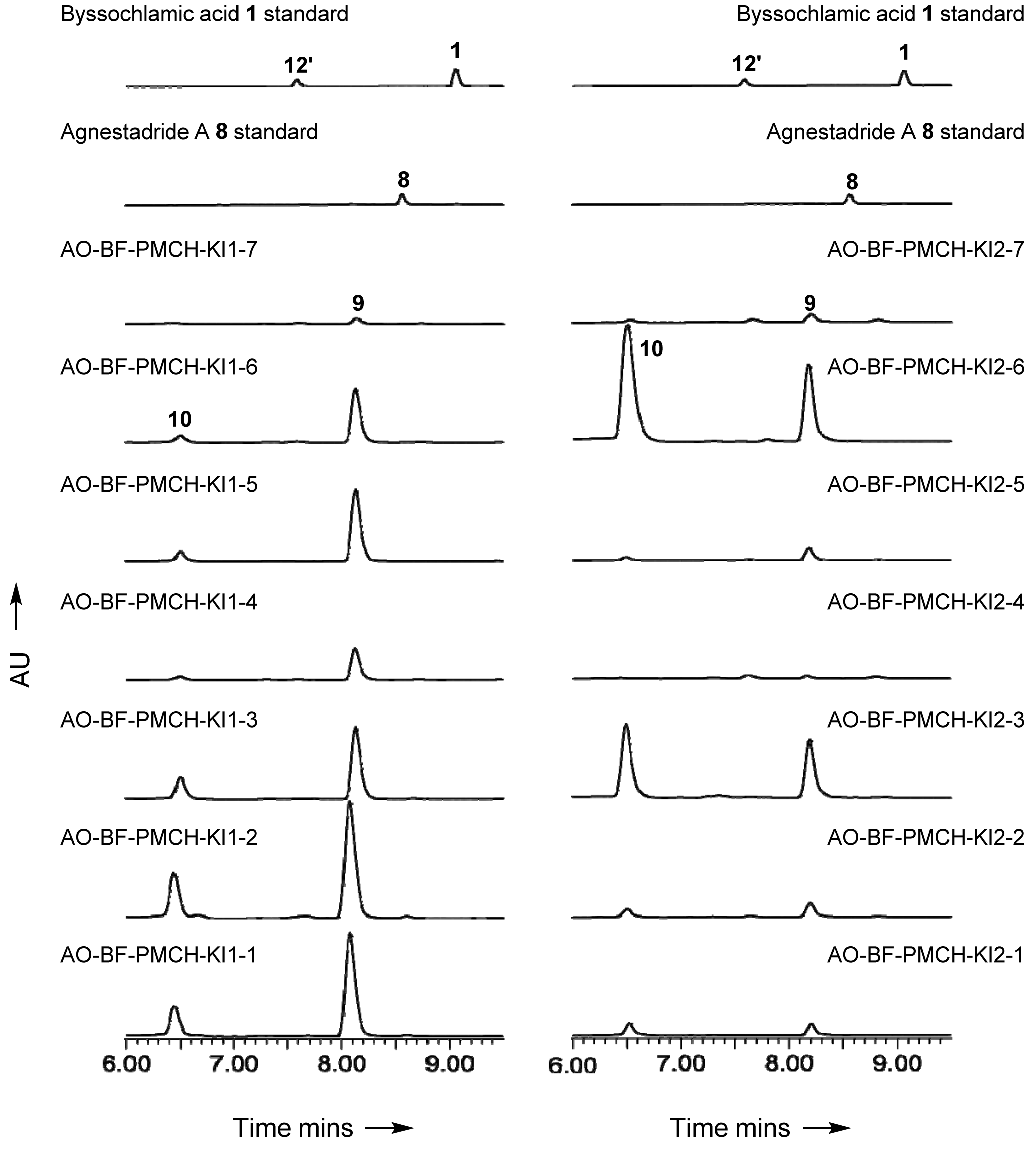

Figure S19: HPLC (DAD) chromatograms comparing the byssochlamic acid **1** and agnestadride A **8** standards to *A. oryzae* strains AO-BF-PMCH+KI1/2. There is no evidence for any dimerized products

13.5.5 LCMS analysis of strain AO-BF-PMCH+KIs+PEBPs

Strain AO-BF-PMCH+KIs expressed the *B. fulva* genes *bfpks1, bfL1, bfL2, bfL3, bfL6, bfL10, bfL5* and *bfL9.* Novel peaks were identified in this strain compared to crude extract from untransformed *A. oryzae* NSAR1. Their UV absorbances and mass fragmentation patterns are shown in Figure S20.

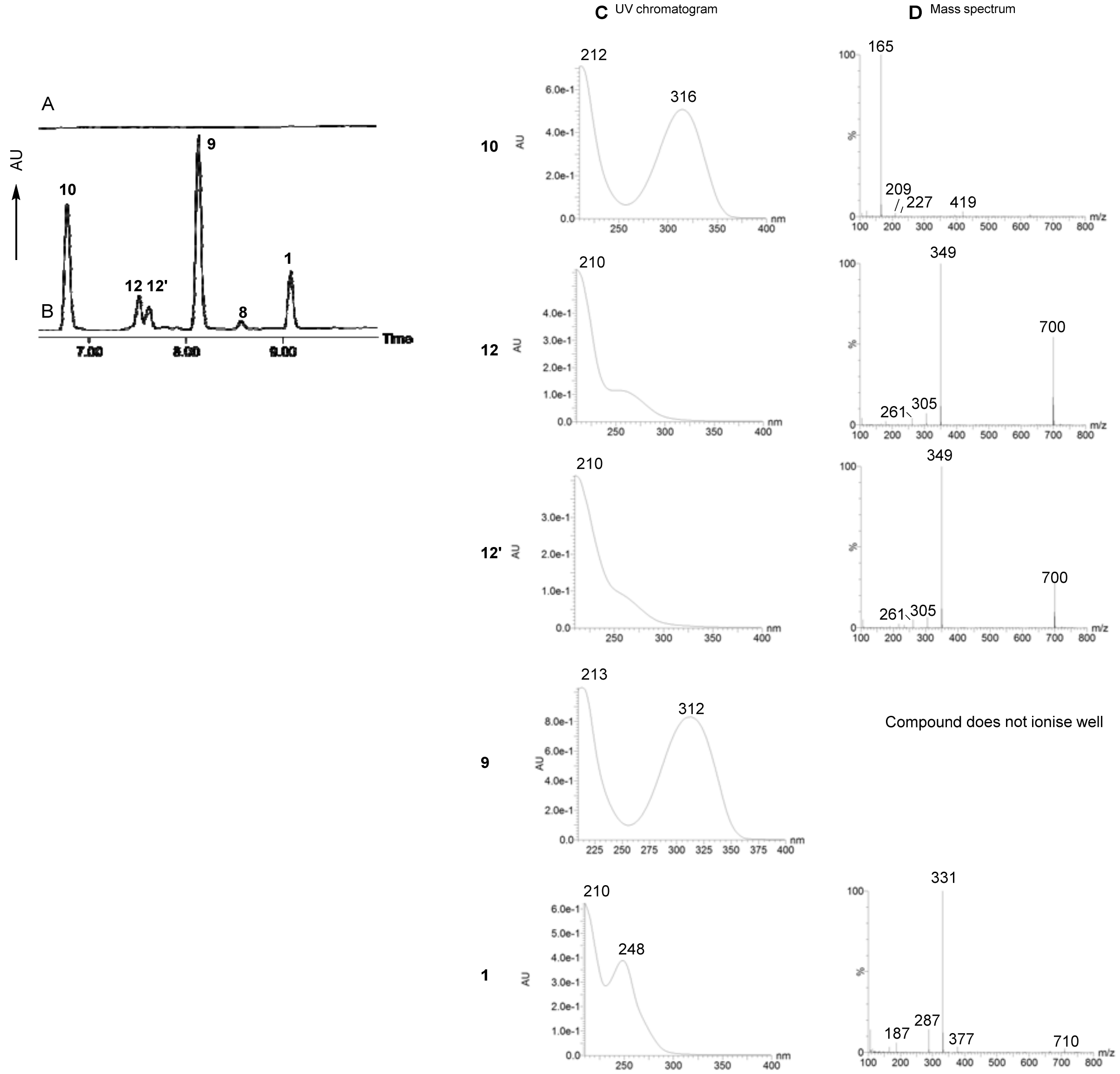

Figure S20: LCMS analysis of strain AO-BF-PMCH+KIs+PEBPs. ***A,*** HPLC (DAD) of crude extract from untransformed *A. oryzae* NSAR1. ***B,*** HPLC (DAD) of crude extract from AO-BF-PMCH+KIs+PEBPs with six novel peaks identified. ***C,*** UV absorbances for compounds **10**, **12**, **12'**, **9**, **8** and **1**. ***D,*** ES- mass fragmentation patterns for compounds **10**, **12**, **12'**, **8** and **1**. Compound **9** does not ionize well

13.5.6 Direct LCMS comparison of strains AO-BF-PMCH+KIs and AO-BF-PMCH+KIs+PEBPs

Equal numbers of spores were inoculated from strains AO-BF-PMCH-KIs and AO-BF-PMCH-KIs-PEBPs into induction medium. 2 replicates for the strain AO-BF-PMCH-KIs and 3 replicates for the strain AO-BF-PMCH-KIs-PEBPs were cultured (100 ml of CMP in a 500 ml flask, 28 °C, 200 rpm shaking for 7 days) and extracted. The crude extracts were dissolved in the same volume of solvent and analysed by LCMS. The integrated area of the UV peak for byssochlamic acid **1** was calculated as follows:

KIsRep1:2181	PEBPsRep1:37714
KIsRep3:806	PEBPsRep2:22684
	PEBPsRep3:41732
**Average = 1493.5**	**Average = 32710**

There is a 21.9 fold increase in production of byssochlamic acid with the inclusion of the PEBPs (Figure S21)

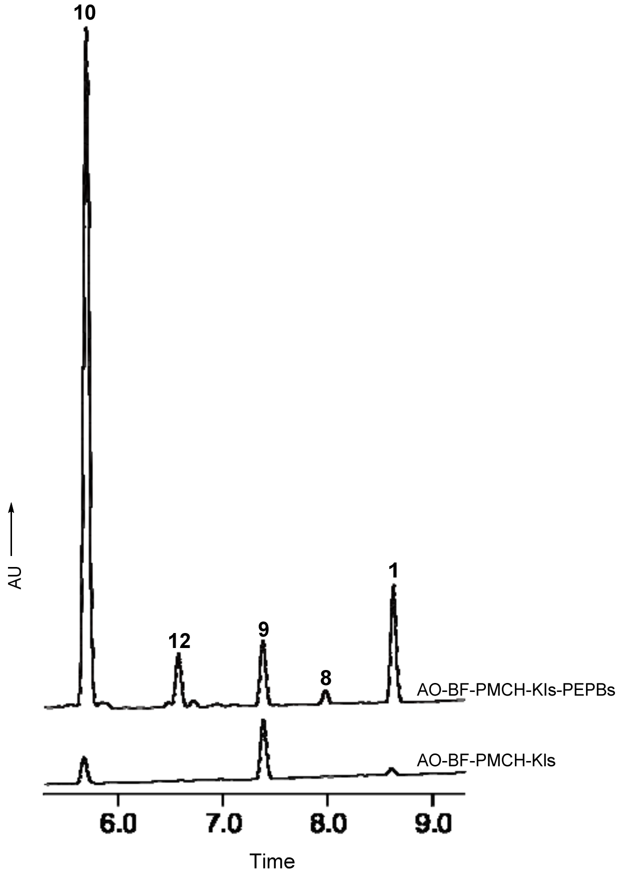

Figure S21: LCMS direct comparison of strains AO-BF-PMCH+KIs and AO-BF-PMCH+KIs+PEBPs.

14. RT-PCR analysis of strains AO-BF-PMCH+KI1 1-7 and AO-BF-PMCH+KI2 1-7

Strains AO-BF-PMCH+KI1 1-7 and AO-BF-PMCH+KI2 1-7 were cultured under induction conditions (100ml of CMP in a 500ml flask, 28 °C, 200 rpm shaking for 7 days). 10 ml of the culture was pelleted and lyophilized for RNA extraction. The remainder of the culture was subjected to metabolite extraction. LCMS analysis confirmed that the monomers **9** and **10** were produced. No evidence for any dimerized products was apparent (Figure S19).
After RNA extraction, cDNA synthesis was performed and RT-PCR analysis was conducted to determine whether the KI1 (*bfL6*) and KI2 (*bfL10*) were being expressed. Primers BF-KI1-F and -R were used to amplify a region of the KI1 gene (*bfL6*) and primers BF-KI2-F and -R were used to amplify a region of the KI2 gene (*bfL10*). In the majority of the strains, it was apparent that the KI genes were being expressed (Figure S22).

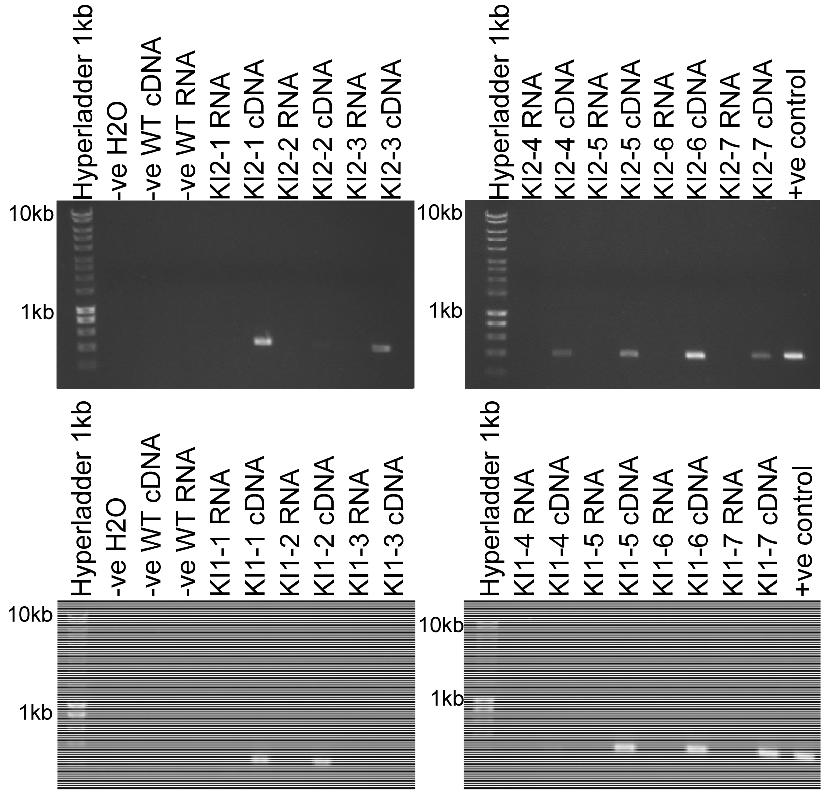

Figure S22: RT-PCR analysis of strains AO-BF-PMCH+KI1 1-7 and AO-BF-PMCH+KI2 1-7.

15. Coexpression of *bfpks1*, *bfL2* and *bfL3*

Plasmid pTYarg-BF-PMCH (Section 10.2) was modified to remove the *bfL1* gene to produce pTYarg-BF-PMC. pTYarg-BF-PMCH was digested with *Bbv*CI which cuts within the gpdA promoter. Primers pTY-F1 and PgpdA-Nd-R were used to amplify a deletion fragment to replace the *bfL1* gene using empty pTYarg-GS plasmid as a template. The digested pTYarg-BF-PMCH and the patch fragment were recombined using homologous recombination in *S. cerevisiae* to produce pTYarg-BF-PMC (Figure S23). This plasmid was transformed into *A. oryzae* NSAR1 and 7 transformants (designated AO-BF-PMC 1-7) were selected. Genomic DNA from all transformants was shown to have incorporated the required genes. All transformants were grown in production media under standard conditions and extracted in the usual way. Compounds **9** and **10** could not be detected by LCMS analysis (Figure S24).

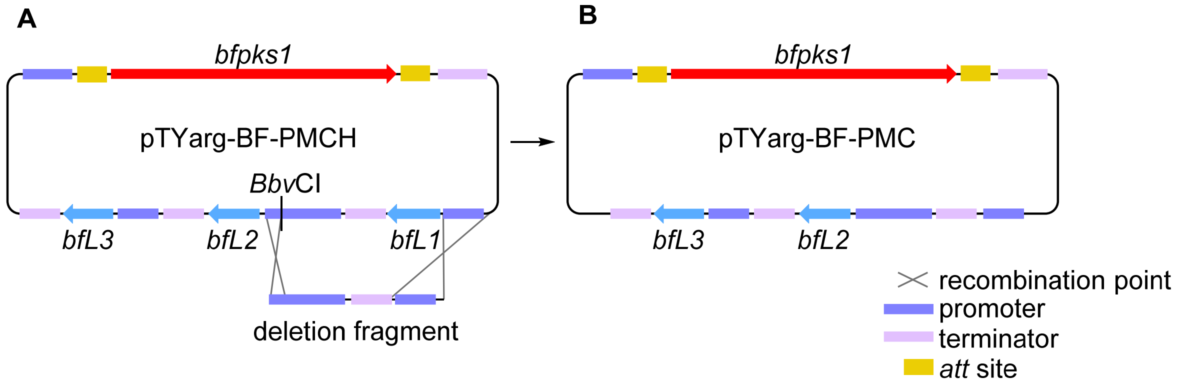

Figure S23: Construction of the plasmid pTYarg-BF-PMC by yeast recombination. ***A,*** pTYarg-BF-PMCH was cut with *Bbv*CI (within the gpdA promoter). A deletion fragment was amplified from pTYarg-GS. ***B,*** Yeast recombination produced plasmid pTYarg-BF-PMC.

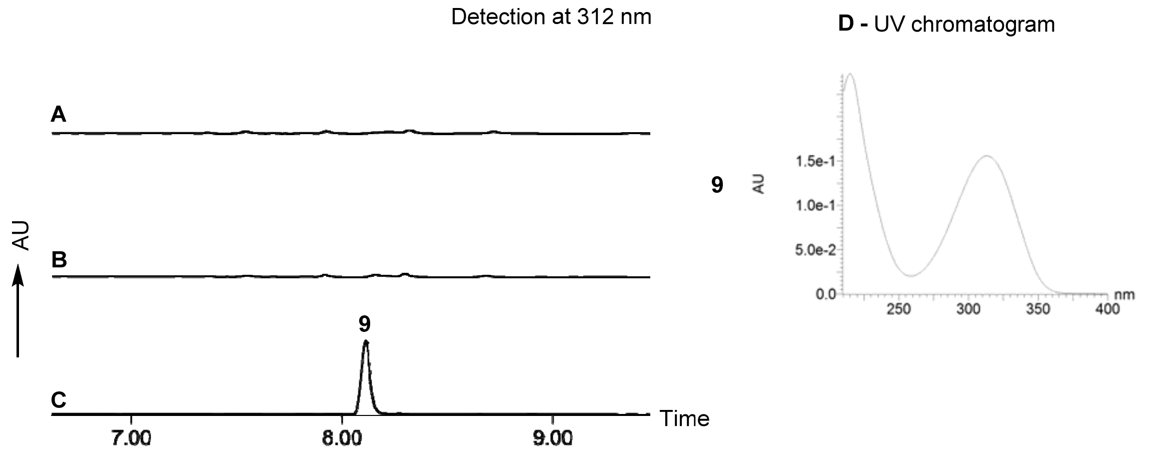

Figure S24: LCMS analysis of strain AO-BF-PMC-1. ***A,*** HPLC (DAD at 312 nm) of crude extract from untransformed *A. oryzae* NSAR1. ***B,*** HPLC (DAD at 312 nm) of crude extract from AO-BF-PMC-1 (all AO-BF-PMC strains 1-7 gave the same phenotype) ***C,*** HPLC (DAD at 312 nm) of crude extract from AO-BF-PMCH. Compound **9** is identified. In this extract no compound **10** was present, likely due to complete conversion to **9**. ***D,*** UV absorbance for compound **9**. No mass fragmentation pattern is shown as **9** does not ionize well.

16. Analysis of purified byssochlamic acid 1 from expression experiments.

Byssochlamic acid **1** was purified using a Waters mass-directed autopurification system comprising of a Waters 2767 autosampler, Waters 2545 pump system, a Phenomenex Kinetex Axia column (5µ, C_{18}, 100 Å, 21.2 × 250 mm) equipped with a Phenomenex Security Guard precolumn (Luna C_5 300 Å) eluted at 20 mL/min at ambient temperature. Solvent A, HPLC grade H_2O + 0.05% formic acid; Solvent B, HPLC grade CH_3CN + 0.045% formic acid. The post-column flow was split (100:1) and the minority flow was made up with HPLC grade MeOH + 0.045% formic acid to 1 mL·min for simultaneous analysis by diode array (Waters 2998), evaporative light scattering (Waters 2424) and ESI mass spectrometry in positive and negative modes (Waters SQD-2). Detected peaks were collected into glass test tubes. Combined tubes were evaporated under a flow of dry N_2 gas, weighed, and residues dissolved directly in NMR solvent for NMR analysis.

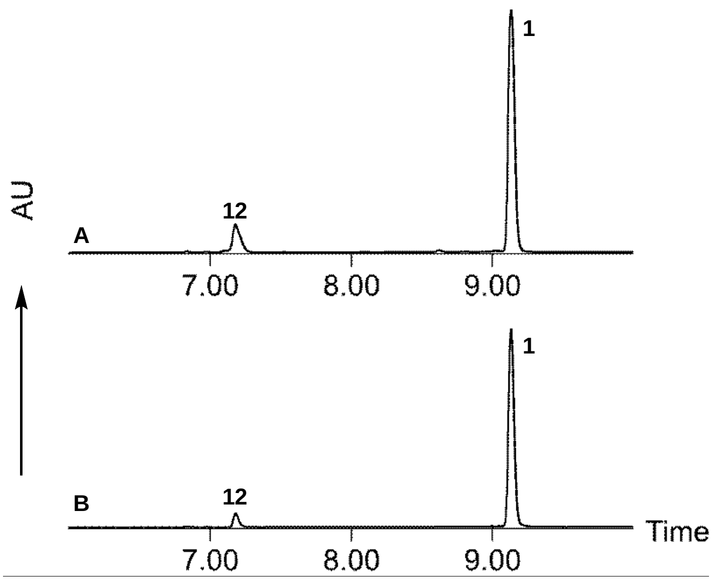

Figure S25: HPLC comparison of byssochlamic acid **1** purified from WT *B. fulva* (**A**) and purified from *A. oryzae* AO-BF-PMCH+KIs+PEBPs (**B**).

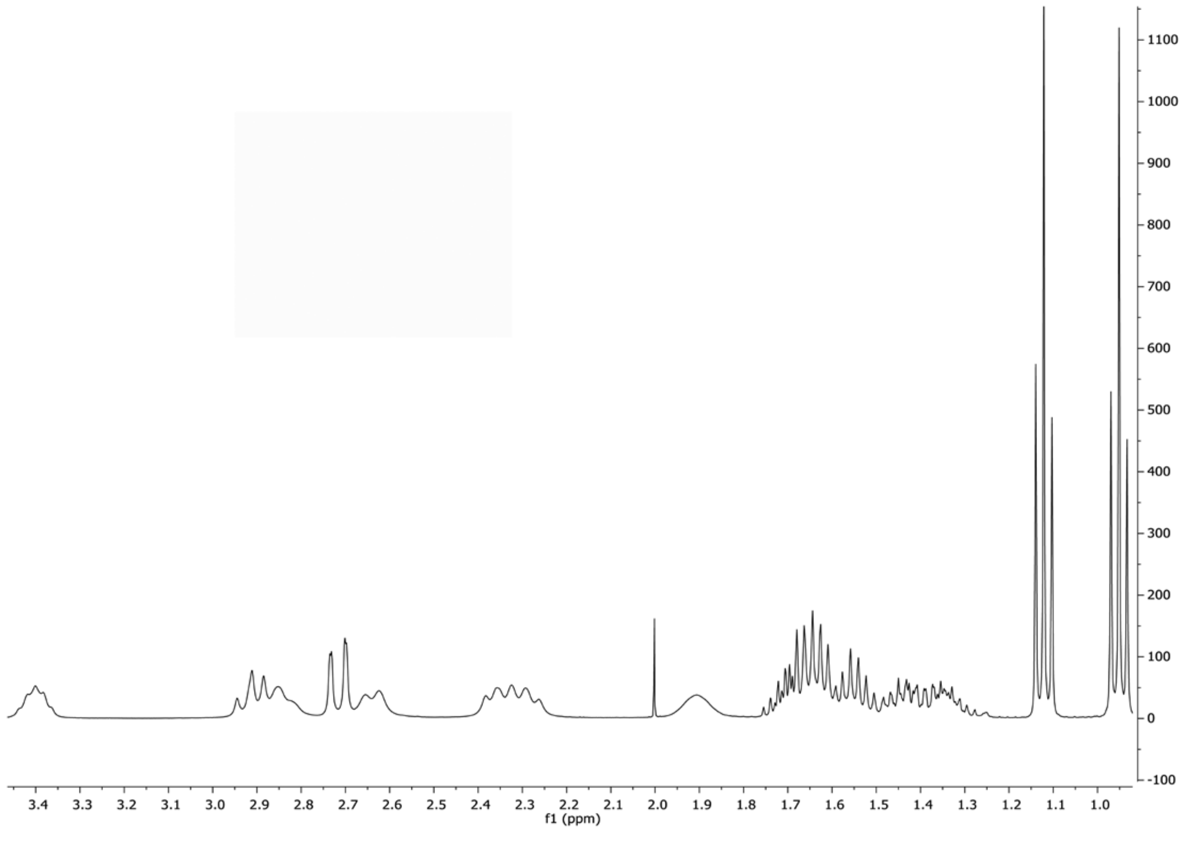

Figure S26: ^{1}H NMR spectrum of byssochlamic acid **1** from *B. fulva* IMI 40021.[51]

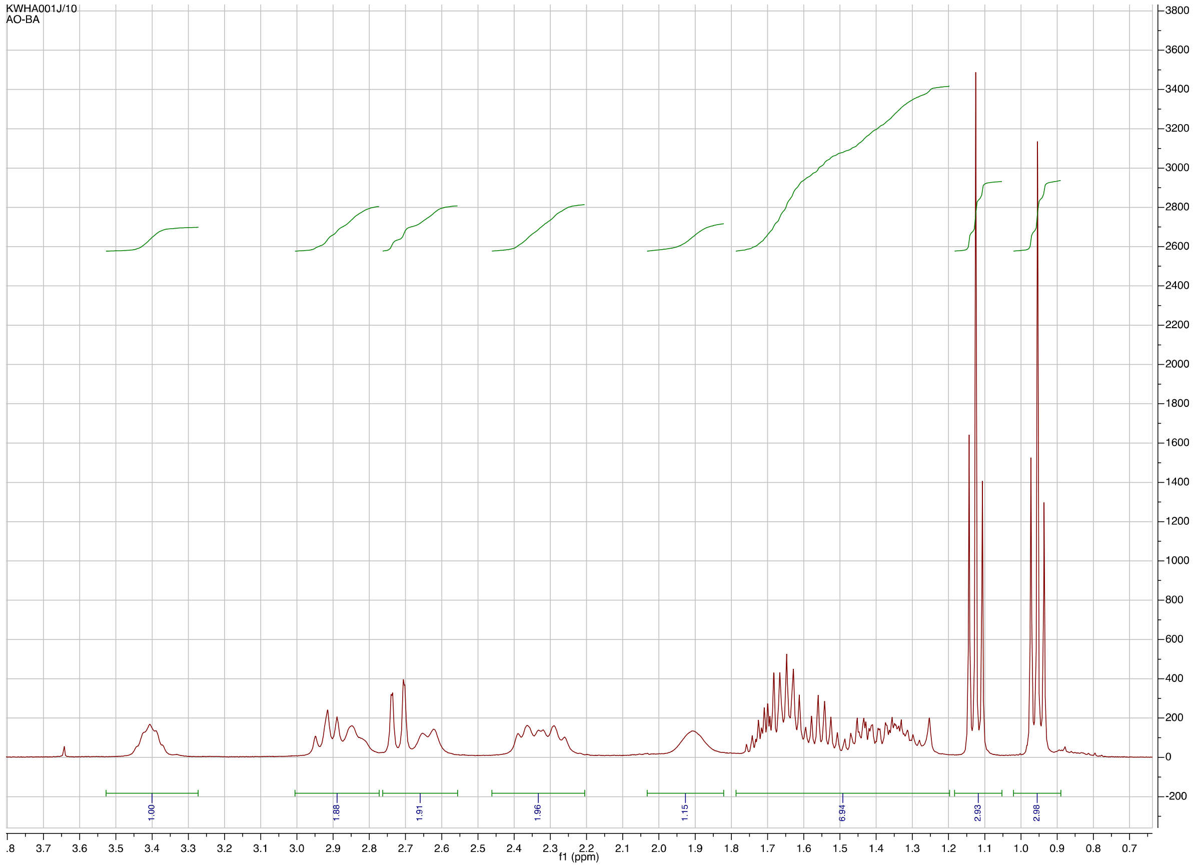

Figure S27: ^{1}H NMR spectrum of byssochlamic acid **1** from *A. oryzae* AO-BF-PMCH+KIs+PEBPs.

17. Stereochemistry of byssochlamic acid 1 from *A. oryzae* AO-BF-PMCH+KIs+PEBPs

B. fulva produces (+)-byssochlamic acid **1**. X-ray crystallography has confirmed the '*cis*' configuration of the two ring-alkyl groups and the 2*R*,7*S* absolute configuration. (-)-byssochlamic acid 1' is also a known compound.[52] White and coworkers have attempted to synthesise the diastereomer *epi*-byssochlamic acid **1***.[53] They calculated a difference in energy between the *cis* **1** and *trans* **1*** diastereomers of 10.9 kJ·mol^{-1}, favouring the *cis* configuration. Attempts to synthesise **1*** always resulted in the production of **1** because of a facile acid catalysed epimerisation at the 7-centre *via* a presumed maleic-itaconic anhydride tautomerisation to the lower energy diastereomer **1**. The $[\alpha]_D$ of byssochlamic acid **1** isolated from *A. oryzae* was + 97.5; The $[\alpha]_D$ of byssochlamic Acid **1** isolated from *B. fulva* is + 108.[54]

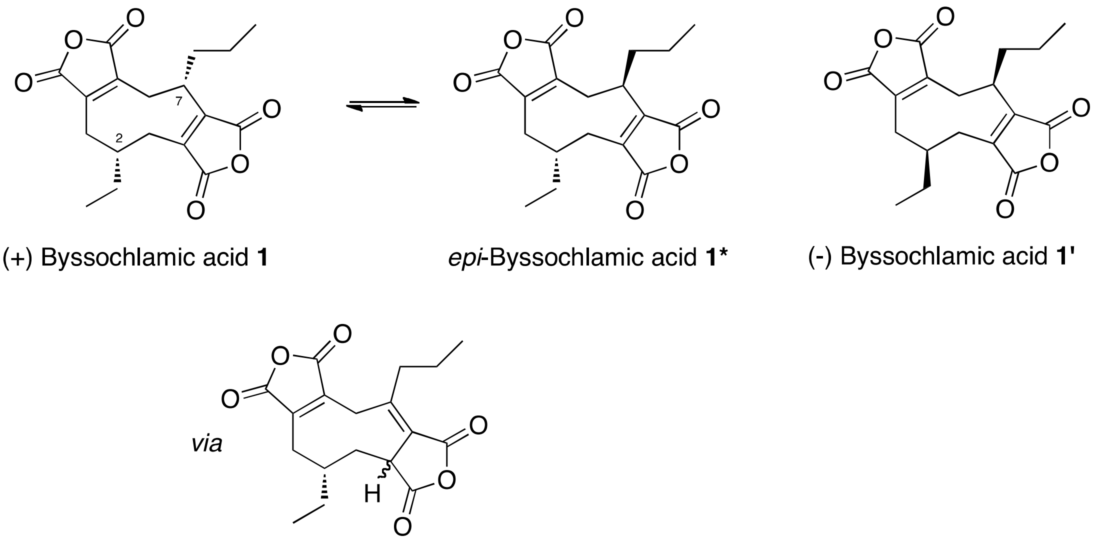

Figure S28: Stereoisomers of **1**.

18. References

[1] J. Sambrook, D. W. Russell, *Molecular Cloning: A Laboratory Manual*, 3rd Edition ed., Cold Spring Harbor Laboratory Press, New York, **2001**.

[2] F. H. Jin, J.-i. Maruyama, P. R. Juvvadi, M. Arioka, K. Kitamoto, *FEMS Microbiol. Lett,*. **2004**, *239*, 79-85.

[3] CABI culture collection. CABI Europe, Bakeham Lane, Egham, Surrey, UK. TW20 9TY.

[4] C. Trapnell, L. Pachter, S. L. Salzberg, *Bioinformatics,* **2009**, *25*, 1105-1111.

[5] V. Solovyev, P. Kosarev, I. Seledsov, D. Vorobyev, *Genome Biol.,* **2006**, *7*, S10.

[6] S. Altschul, T. Madden, A. Schaffer, J. Zhang, Z. Zhang, W. Miller, D. Lipman, *Nucleic Acids Res,*. **1997**, *25*, 3389 - 3402.

[7] S. Hunter, P. Jones, A. Mitchell, R. Apweiler, T. K. Attwood, A. Bateman, T. Bernard, D. Binns, P. Bork, S. Burge, E. de Castro, P. Coggill, M. Corbett, U. Das, L. Daugherty, L. Duquenne, R. D. Finn, M. Fraser, J. Gough, D. Haft, N. Hulo, D. Kahn, E. Kelly, I. Letunic, D. Lonsdale, R. Lopez, M. Madera, J. Maslen, C. McAnulla, J. McDowall, C. McMenamin, H. Mi, P. Mutowo-Muellenet, N. Mulder, D. Natale, C. Orengo, S. Pesseat, M. Punta, A. F. Quinn, C. Rivoire, A. Sangrador-Vegas, J. D. Selengut, C. J. A. Sigrist, M. Scheremetjew, J. Tate, M. Thimmajanarthanan, P. D. Thomas, C. H. Wu, C. Yeats, S.-Y. Yong, *Nucleic Acids Res.,* **2012**, *40*, 4725-4725.

[8] J. Kennedy, K. Auclair, S. G. Kendrew, C. Park, J. C. Vederas, C. R. Hutchinson, *Science,* **1999**, *284*, 1368-1372.

[9] S. M. Baxter, J. S. Rosenblum, S. Knutson, M. R. Nelson, J. S. Montimurro, J. A. Di Gennaro, J. A. Speir, J. J. Burbaum, J. S. Fetrow, *Mol. Cell. Proteomics,* **2004**, *3*, 209-225.

[10] Y. C. Huang, Y. H. Chen, S. R. Lo, C. I. Liu, C. W. Wang, W. T. Chang, *Mol. Microbiol.,* **2004**, *53*, 81-91.

[11] T. Tabuchi, H. Aoki, H. Uchiyama, T. Nakahara, *Agric. Biol. Chem.,* **1981**, *45*, 2823-2829.

[12] R. C. Vargas, S. Tenreiro, M. C. Teixeira, A. R. Fernandes, I. Sa-Correia, *Antimicrob. Agents Chemother.,* **2004**, *48*, 2531-2537.

[13] X. Wang, N. Li, B. Liu, H. Sun, T. Chen, H. Li, J. Qiu, L. Zhang, T. Wan, X. Cao, *J. Biol. Chem.,* **2004**, *279*, 45855-45864.

[14] P. K. Chang, J. W. Cary, D. Bhatnagar, T. E. Cleveland, J. W. Bennett, J. E. Linz, C. P. Woloshuk, G. A. Payne, *Appl. Environ. Microbiol.,* **1993**, *59*, 3273-3279.

[15] D. R. Fitzpatrick, E. Germain-Lee, D. Valle, *Genomics,* **1995**, *27*, 457-466.

[16] H. Haas, M. Schoeser, E. Lesuisse, J. F. Ernst, W. Parson, B. Abt, G. Winkelmann, H. Oberegger, *Biochem. J.,* **2003**, *371*, 505-513.

[17] H. M. ElBerry, M. L. Majumdar, T. S. Cunningham, R. A. Sumrada, T. G. Cooper, *J. Bacteriol.,* **1993**, *175*, 4688-4698.

[18] K. Zhou, P. R. G. Brisco, A. E. Hinkkanen, G. B. Kohlhaw, *Nucleic Acids Res.,***1987**, *15*, 5261-5273.

[19] S. Sato, H. Suzuki, U. Widyastuti, Y. Hotta, S. Tabata, *Curr. Genet.,* **1994**, *26*, 31-37.

[20] R. J. Cox, *Org. Biomol. Chem.,* **2007**, *5*, 2010-2026.

[21] S. Kauppinen, M. Siggaard-Andersen, P. von-Wettstein-Knowles, *Carlsberg Res. Commun.,* **1988**, *53*, 357-370.

[22] S. Poust, I. Yoon, P. D. Adams, L. Katz, C. J. Petzold, J. D. Keasling, *PLoS One,* **2014**, *9,* e109421.

[23] K. L. Kavanagh, H. Jornvall, B. Persson, U. Oppermann, *Cell. Mol. Life Sci,*. **2008**, *65*, 3895-3906.

[24] C. J. Sigrist, E. de Castro, L. Cerutti, B. A. Cuche, N. Hulo, A. Bridge, L. Bougueleret, I. Xenarios, *Nucleic Acids Res.,* **2013**, *41*, D344-347.

[25] G. Yang, M. S. Rose, B. G. Turgeon, O. C. Yoder, *Plant Cell,* **1996**, *8*, 2139-2150.

[26] R. M. Kagan, S. Clarke, *Arch. Biochem. Biophys.,* **1994**, *310*, 417-427.

[27] S. Remington, G. Wiegand, R. Huber, *J. Mol. Biol.,* **1982**, *158*, 111-152.

[28] O. Emanuelsson, H. Nielsen, S. Brunak, G. von Heijne, *J. Mol. Biol.,* **2000**, *300*, 1005-1016.

[29] B. W. Park, K. H. Han, C. Y. Lee, C. H. Lee, P. J. Maeng, *Mol. Cells,* **1997**, *7*, 290-295.

[30] M. Brock, R. Fischer, D. Linder, W. Buckel, *Mol. Microbiol.,* **2000**, *35*, 961-973.

[31] T. Ferea, E. T. Contreras, T. Oung, E. J. Bowman, B. J. Bowman, *Mol. Gen. Genet.,* **1994**, *242*, 105-110.

[32] C. Maerker, M. Rohde, A. A. Brakhage, M. Brock, *FEBS J.,* **2005**, *272*, 3615-3630.

[33] A. R. Horswill, J. C. Escalante-Semerena, *Biochemistry,* **2001**, *40*, 4703-4713.

[34] L. A. Kelley, M. J. E. Sternberg, *Nat. Protoc.,* **2009**, *4*, 363-371.

[35] B. Lohkamp, B. Baeuerle, P.-G. Rieger, G. Schneider, *J. Mol. Biol.,* **2006**, *362*, 555-566.

[36] M. Brock, C. Maerker, A. Schutz, U. Volker, W. Buckel, *Eur. J. Biochem.,* **2002**, *269*, 6184-6194.

[37] M. Kotowska, K. Pawlik, A. Smulczyk-Krawczyszyn, H. Bartosz-Bechowski, K. Kuczek, *Appl. Environ. Microbiol.,* **2009**, *75*, 887-896.

[38] L. Serre, B. Vallée, N. Bureaud, F. Schoentgen, C. Zelwer, *Structure,* **1998**, *6*, 1255-1265.

[39] P. C. Simister, M. J. Banfield, R. L. Brady, *Acta Crystallogr., Sect. D: Biol. Crystallogr.,* **2002**, *58*, 1077-1080.

[40] T. J. Carver, K. M. Rutherford, M. Berriman, M. A. Rajandream, B. G. Barrell, J. Parkhill, *Bioinformatics,* **2005**, *21*, 3422-3423.

[41] F. Sievers, A. Wilm, D. Dineen, T. J. Gibson, K. Karplus, W. Li, R. Lopez, H. McWilliam, M. Remmert, J. Soeding, J. D. Thompson, D. G. Higgins, *Mol. Syst. Biol.,* **2011**, *7*, 539.

[42] K. A K Pahirulzaman, K. Williams, C. M. Lazarus, *Methods Enzymol.,* **2012**, *517*, 241-260.

[43] J. Hartley, Temple, G. F. Brasch, M. A., *Genome Res.,* **2000**, *10*, 1788-1795.

[44] T. Akao, M. Sano, O. Yamada, T. Akeno, K. Fujii, K. Goto, S. Ohasi-Kunihiro, K. Takase, M. Yasukawa-Watanabe, K. Yamaguchi, Y. Kurihara, J.-i. Maruyama, P. R. Juvvadi, A. Tanaka, Y. Hata, Y. Koyama, S. Yamaguchi, N. Kitamoto, K. Gomi, K. Abe, M. Takeuchi, T. Kobayashi, H. Horiuchi, K. Kitamoto, Y. Kashiwagi, M. Machida, O. Akita, *DNA Res.,* **2007**, *14*, 47-57.

[45] P. J. Punt, M. A. Dingemanse, A. Kuyvenhoven, R. D. M. Soede, P. H. Pouwels, C. A. M. J. J. van den Hondel, *Gene,* **1990**, *93*, 101-109.

[46] S. Tada, K. Gomi, K. Kitamoto, K. Takahashi, G. Tamura, S. Hara, *Mol. Gen. Genet.,* **1991**, *229*, 301-306.

[47] C. M. Lazarus, K. Williams, A. M. Bailey, *Nat. Prod. Rep.,* **2014**, *31*, 1339-1347.

[48] R. D. Gietz, R. H. Schiestl, A. R. Willems, R. A. Woods, *Yeast,* **1995**, *11*, 355-360.

[49] M. L. Nielsen, L. Albertsen, G. Lettier, J. B. Nielsen, U. H. Mortensen, *Fungal Genet. Biol.,* **2006**, *43*, 54-64.

[50] P. J. Punt, R. P. Oliver, M. A. Dingemanse, P. H. Pouwels, C. Vandenhondel, *Gene,* **1987**, *56*, 117-124.

[51] A. J. Szwalbe, K. Williams, D. E. O'Flynn, A. M. Bailey, N. P. Mulholland, J. L. Vincent, C. L. Willis, R. J. Cox, T. J. Simpson, *Chem. Commun.,* **2015**, *51*, 17088-17091.

[52] C. Li, R. Yang, Y. Lin, S. Zhou, *Chem. Nat. Compd.*, **2006**, *42*, 290–293.

[53] J. D. White, J. Kim, N. E. Drapela, *J. Am. Chem. Soc.,* **2000**, *122*, 8665–8671.

[54] H. Raistrick and G. Smith, *Biochem. J.*, **1933**, *27*, 1814–1819.
